# *GW* Plus Cumulant Approach for Predicting Core-Level Shakeup Satellites in Large Molecules

**DOI:** 10.1021/acs.jctc.4c01754

**Published:** 2025-03-03

**Authors:** Jannis Kockläuner, Dorothea Golze

**Affiliations:** Faculty of Chemistry and Food Chemistry, Technische Universität Dresden, 01062 Dresden, Germany

## Abstract

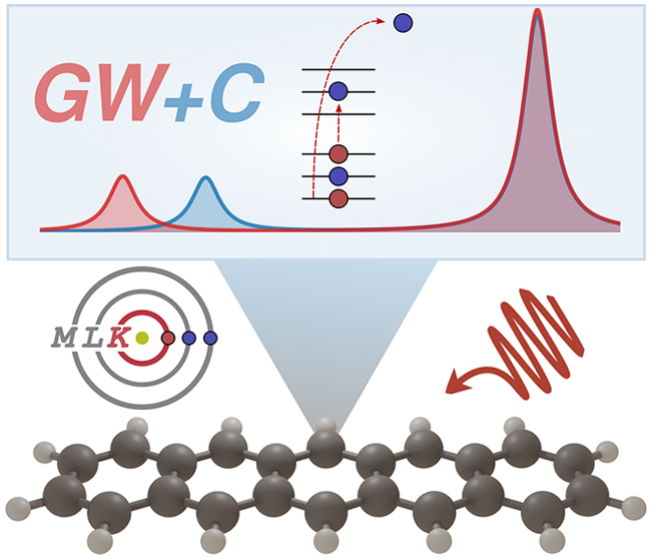

Recently, the *GW* approach has emerged as a valuable tool for computing deep core-level binding energies as measured in X-ray photoemission spectroscopy. However, *GW* fails to accurately predict shakeup satellite features, which arise from charge-neutral excitations accompanying the ionization. In this work, we extend the *GW* plus cumulant (*GW* + *C*) approach to molecular 1s excitations, deriving conditions under which *GW* + *C* can be reliably applied to shakeup processes. We present an efficient implementation with *O*(*N*^4^) scaling with respect to the system size *N*, within an all-electron framework based on numeric atom-centered orbitals. We demonstrate that decoupling the core and valence spaces is crucial when using localized basis functions. Additionally, we meticulously validate the basis set convergence of the satellite spectrum for 65 spectral functions and identify the importance of diffuse augmenting functions. To assess the accuracy, we apply our *GW* + *C* scheme to π-conjugated molecules containing up to 40 atoms, predicting dominant satellite features within 0.5 eV of experimental values. For the acene series, from benzene to pentacene, we demonstrate how *GW* + *C* provides critical insights into the interpretation of experimentally observed satellite features.

## 1 Introduction

X-ray photoelectron spectroscopy (XPS) probes strongly localized deep core-levels and is routinely used for chemical analysis.^1−3^ In XPS spectra, satellite features appear alongside the main lines, which correspond to core-level binding energies. We distinguish between shake-off, plasmon, or shakeup satellites. Shake-offs are additional charged excitations accompanying the photoionization process, giving rise to broad spectral features. Plasmons are collective electronic oscillations predominantly found in solid-state systems, while shakeup excitations involve coupled electron–hole pairs and are typically observed in molecular spectra. However, for large molecules the distinction between the two phenomena can be difficult.^4^ In this work we focus on shakeup satellites. Particularly strong shakeup features have been reported in transition metal complexes and in molecules containing large π-electron systems.^5−7^

Shakeup excitations involve coupled many-electron interactions, requiring an explicit treatment of correlation effects. These effects are not captured by mean-field methods like Hartree–Fock (HF) or Kohn–Sham density functional theory (DFT).^7,8^ Instead, various correlated methods have been used to simulate shakeup satellites. Cederbaum and co-workers pioneered the use of Green’s function methods for molecular systems, applying techniques like the algebraic diagrammatic construction (ADC) scheme^9−11^ and the two-particle-hole Tamm-Dancoff approximation (2ph-TDA) to study valence- and core-level ionization spectra of small molecules, though with mixed success.^12−14^

Wave function-based approaches have proven to be highly accurate for modeling shakeup processes, including configuration interaction,^15−19^ symmetry-adapted cluster configuration interaction (SAC–CI)^20−22^ and quasi-degenerate perturbation theory configuration interaction (QDPTCI).^23−25^ In addition, coupled-cluster techniques like (real-time) equation-of-motion coupled cluster Green’s function approaches^26−28^ have been developed, using a mix of wave function and propagator techniques. Despite the variety of methodologies available, accurate *ab initio* simulations for shakeup satellites have only been reported for small molecular systems, such as water,^13,27,29^ carbon monoxide,^25,30−32^ and formaldehyde.^33−35^

As an alternative, the *GW* approximation, introduced by Hedin in 1965,^36^ offers a framework for the computation of the one-particle propagator, including correlation effects in a perturbative way.^37−39^ The *GW* method is a powerful and efficient tool for predicting ionization energies and is widely regarded as the gold standard for simulating photoemission spectra in both solid-state^39^ and, more recently, molecular systems.^40,41^ While the *GW* method excels at predicting ionization energies, it falls short for satellite features, predicting too few satellites and with incorrect energies.^38,42,43^ This limitation of *GW* is attributed to the neglect of vertex corrections, necessitating approaches beyond *GW* for accurately simulating shakeup satellites.^38^

A systematic way to add vertex corrections for the satellites is to combine the *GW* self-energy with a cumulant *ansatz* for the propagator, resulting in the *GW* + *C* approximation. The *GW* + *C* method has been successfully applied to plasmonic satellites in the valence spectra of various materials, such as sodium,^44−48^ aluminum,^44,49−51^ silicon,^52−56^ graphene^57,58^ and more,^51,59^ showing good agreement with experiment. Despite its success, we are aware of only one attempt to apply the *GW* + *C* approach to molecules, targeting the valence states of benchmark systems like methane, water and neon.^60^ In this work, we advance the *GW* + *C* approach for the computation of satellites in deep core-level spectra of molecular systems.

The application of *GW* to deep core-levels is a rather recent development.^61−75^ We showed that the following points are important for core-level *GW* calculations: The complex structure of the self-energy in the core region demands the use of a highly accurate technique for the frequency integration, e.g., the contour deformation (CD) approach or an fully analytical approach.^61−63^ Furthermore, either self-consistency in *G* or a large amount of exact exchange in the DFT starting point is required to ensure a distinct quasiparticle (QP) solution, as the one-shot scheme shifts the QP into the satellite region, causing the satellites to gain artificial spectral weight.^62,63^

Core-level *GW* calculations with CD scale *O*(*N*^5^) with respect to system size *N*.^61^ We recently proposed the CD-WAC method (CD with *W* analytic continuation),^76^ achieving a scaling reduction to *O*(*N*^4^) for core-levels, while retaining the CD accuracy. In addition, we introduced the *G*_Δ*H*_*W*_0_ variant, which mimics eigenvalue self-consistency in *G* with a constant Hedin shift Δ*H. G*_Δ*H*_*W*_0_ effectively restores the QP peak,^63^ at a fraction of the computational cost of a partial self-consistent scheme.

Building on our core-level *GW* developments,^61−63,76^ we introduce a *GW* + *C* scheme within an all-electron framework using localized basis functions. This work reports the first application of *GW* + *C* to molecular shakeup satellites accompanying 1s excitations. We derive conditions for applying *GW* + *C* to shakeup processes, along with numerical settings that include recommendations on basis sets, starting points, and other factors affecting numerical stability. To the best of our knowledge, we also present the largest full *ab initio* calculations of core-level shakeup satellites to date, applying our *GW* + *C* approach to organic molecules with up to 40 atoms.

The paper is structured as follows: In Sections 2 and 3, we summarize the key equations of *GW* and *GW* + *C* theory for deep core-levels. In Section 4, we present the final working equations and implementation together with a brief overview of the low-scaling CD-WAC method. The relevant technical settings are summarized in Section 5. In Section 6, we provide a thorough discussion of technical aspects of *GW* + *C*. We then assess the accuracy of the method by comparing the *GW* + *C* predictions to experiment and *GW*. Finally, we summarize our key findings and give an outlook on future developments in Section 7.

## 2 *GW* Theory

### 2.1 *G*_0_*W*_0_ Approximation

The *GW* approximation is a popular many-body perturbation theory approach derived from Hedin’s equations by neglecting all vertex corrections.^36−39^ In *G*_0_*W*_0_, the *GW* solution is approximated by using only the first iteration of the *GW* equations starting from a mean-field propagator *G*_0_(ω), e.g., based on a DFT or HF reference. In the time-ordered formalism, the Lehmann representation of *G*_0_(ω) is defined as
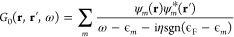
1Here, ϵ_F_ is the Fermi level and η is an infinitesimal broadening parameter. *G*_0,*n*_(ω) = ⟨ψ_*n*_|*G*_0_(**r**, **r**′, ω)|ψ_*n*_⟩ is diagonal in the basis of molecular orbitals ψ_*n*_(**r**), where *n* is the index of a molecular orbital with an energy ϵ_*n*_.^39^
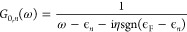
2The molecular orbitals are expanded in a finite set of atomic orbitals φ_*m*_
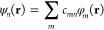
3where *c*_*mn*_ are expansion coefficients determined by a mean-field calculation. In the following, we use the common notation that arbitrary molecular orbitals are indexed as *m*, *n*, while occupied (unoccupied) orbitals are specified by indices *i*, *j* (*a*, *b*).

The central quantity in *G*_0_*W*_0_ is the dynamic self-energy Σ^*G*_0_*W*_0_^(ω), which is computed from a mean-field propagator *G*_0_ and the screened Coulomb interaction *W*_0_. In the basis of molecular orbitals, the diagonal elements Σ_*n*_^*G*_0_*W*_0_^(ω) are given by
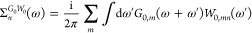
4The diagonal elements of the screened Coulomb interaction *W*_0,*mn*_≔*W*_0,*mnmn*_ are computed in the random-phase approximation (RPA). The correlation part *W*^*c*^ = *W*–*v* is given by
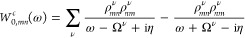
5where *v* denotes the bare Coulomb interaction. Ω^ν^ and ρ_*mn*_^ν^ are the RPA excitations energies and transition moments.

The RPA excitation energies are obtained as solution of an eigenvalue equation in the subspace of single particle excitations from occupied orbitals *i* to virtual orbitals *a*.
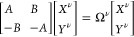
6The matrices *A* and *B* couple singly excited states and are defined in eq 7.
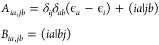
7The integrals (*ia*|*jb*) are the standard two-electron Coulomb repulsion integrals in chemists’ notation. The transition moments ρ_*mn*_^ν^ are computed from the excitation vectors *X*^ν^,*Y*^ν^.
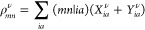
8Using eqs 2 and 5, the frequency integral in eq 4 is obtained in the form that we will refer to as fully analytical approach. The self-energy is split in a static exchange part Σ_*n*_^*x*, *G*_0_*W*_0_^ and a frequency-dependent correlation part Σ_*n*_^*c*,*G*_0_*W*_0_^(ω).
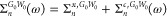
9
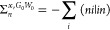
10
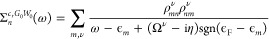
11The interacting propagator *G*_*n*_ is obtained from a Dyson equation:
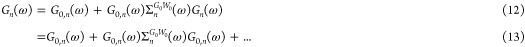
By inverting eq 12 and inserting eq 2, the following compact expression for the propagator is derived
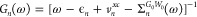
14where *v*_*n*_^*xc*^ = ⟨ψ_*n*_|*v*^*xc*^|ψ_*n*_⟩ is the approximate mean-field exchange–correlation potential which has to be removed to avoid double-counting of exchange- and correlation effects.

The poles of *G*_*n*_(ω) are the charged excitations of the system within the *G*_0_*W*_0_ approximation and can be directly linked to the experiment using the spectral function *A*_*n*_^*G*_0_*W*_0_^(ω).
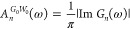
15By inserting eq 14 in eq 15 and dividing the self-energy in its real- and imaginary part, the spectral function can be rewritten as
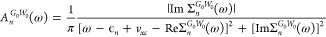
16The main peaks in eq 15 appear at the QP energies ϵ_*n*_^QP^. The spectral weight of the QP peaks is large, as Im Σ_*n*_^G_0_*W*_0_^ is typically small in this region.

Instead of evaluating *A*_*n*_^*G*_0_*W*_0_^(ω) over a broad frequency range, ϵ_*n*_^QP^ can be obtained as solution of a nonlinear equation.
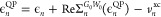
17

Equation 17 is solved iteratively, which typically takes around 10 steps and is computationally much cheaper compared to the computation of *A*_*n*_^*G*_0_*W*_0_^(ω) over a large frequency range.

### 2.2 Satellites in the *G*_0_*W*_0_ Approximation

**Figure 1:**
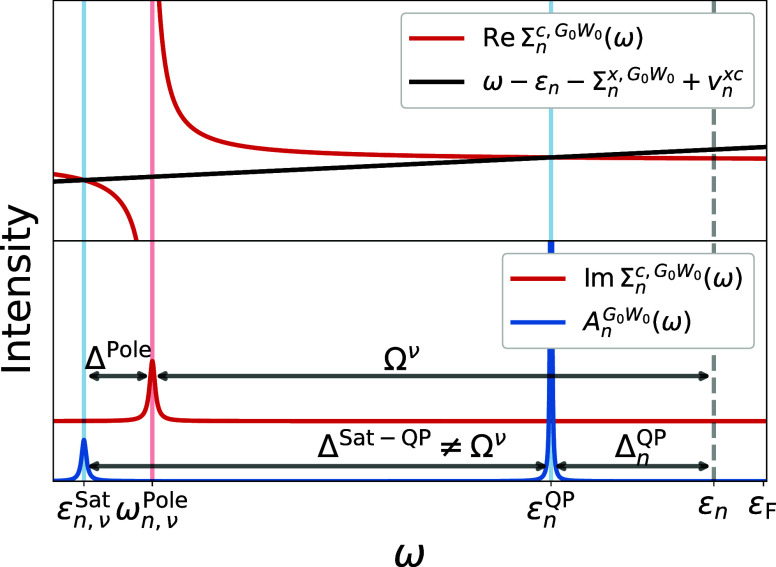
Sketch of the *G*_0_*W*_0_ solution for a system with a single satellite. The upper part shows the approximate graphical solution of eq 17 (see ref (39)), with the two solutions highlighted in blue. The bottom part displays the resulting spectral function along with Im Σ_*n*_^*c*,*G*_0_*W*_0_^(ω).

The *G*_0_*W*_0_ spectral function exhibits QP peaks corresponding to the ionization process, along with additional satellite features of plasmonic or shakeup character. Satellites are expected to appear roughly at ϵ_*n*_^QP^ – Ω^ν^ because these satellites result from charge-neutral excitations Ω^ν^ coupling to the ionization process. *G*_0_*W*_0_ provides a poor qualitative and quantitative description of the satellites. To facilitate the discussion, we plot in Figure 1 the real and imaginary parts of Σ_*n*_^*c*,*G*_0_*W*_0_^(ω), along with the spectral function *A*_*n*_^*G*_0_*W*_0_^(ω), for a system featuring one QP peak and one satellite.

First, the *G*_0_*W*_0_ satellites are not defined relative to the QP peak positions ϵ_*n*_^QP^, but relative to the DFT positions ϵ_*n*_. This is because in *G*_0_*W*_0_, satellites occur near frequencies where the real part of Σ^*c*^ has poles, and its imaginary part exhibits complementary peaks, see Figure 1. If *n* corresponds to an occupied level, these poles in ReΣ_*n*_^*c*^ are located at ω_*n*,ν_^Pole^ = ϵ_*n*_ – Ω^ν^ rather than ϵ_*n*_^QP^ – Ω^ν^. Using a functional based on the generalized gradient approximation (GGA) or a hybrid functional with low amount of exact exchange as starting point, the QP correction Δ_*n*_^QP^ = ϵ_*n*_^QP^ – ϵ_*n*_ is negative and the satellites are generally too close to the QP peak.

Second, the satellites in the spectral function do not occur at ω_*n*,ν_^Pole^ = ϵ_*n*_ – Ω^ν^, but at more negative frequencies ϵ_*n*,ν_^Sat^, as shown in the lower part of Figure 1. In other words, peaks in Im Σ_*n*_^*c*,*G*_0_*W*_0_^ do not directly translate into peaks in *A*_*n*_^*G*_0_*W*_0_^. This can be seen in eq 16, where the denominator introduces an offset, labeled as Δ^Pole^ in Figure 1. Consequently, the interpretation of a charge-neutral excitation coupling to an ionization—while still present in the self-energy, albeit with an incorrect reference—is lost in the spectral function.

Third, the satellites in *G*_0_*W*_0_ contain only single RPA excitations, while many-electron excitations require specific vertex corrections. As a result, it has been shown for solid-state systems that *G*_0_*W*_0_ does not produce the expected series of plasmonic satellites but instead yields a spurious single “plasmaron”.^42,44,52,77^ In Section 3.2, we will demonstrate that *G*_0_*W*_0_ also generates too few peaks in the shakeup case.

In summary, *G*_0_*W*_0_ predicts too few satellites at too negative frequencies (or equivalently too high binding energies) with a satellite QP-splitting of Δ^Sat–QP^ = ϵ_*n*_^QP^ – ϵ_*n*,ν_^Sat^ = Ω – |Δ_*n*_^QP^| + |Δ^Pole^| with generally |Δ_*n*_^QP^| > |Δ^Pole^| for core-levels.

### 2.3 *GW* for Core-Levels: Beyond *G*_0_*W*_0_

We demonstrated that *G*_0_*W*_0_, when starting from a GGA reference such as the Perdew–Burke–Ernzerhof (PBE)^78^ functional, fails to produce a unique QP solution for molecular 1s excitations.^62,63^ Instead, multiple solutions with equal spectral weight are obtained.^62^ This is in striking contrast to experimental results,^79^ which show a strong 1s main excitation. For small molecules, the intensities of the satellite features are typically orders of magnitude smaller compared to the main line.^29,30,80^

The failure of *G*_0_*W*_0_@PBE for deep core-levels arises from the incorrect positioning of the satellites. While |Δ_*n*_^QP^| typically ranges from 1–2 eV for valence levels,^39^ it increases to 20–35 eV for 1s excitations of second-row elements.^61^ For core-levels, |Δ_*n*_^QP^| can approach or even exceed Ω^ν^. In the former case, the satellite-QP separation becomes very small, whereas in the latter, satellites may appear at lower binding energies (more positive frequencies) than the QP peak—or, in other words, the QP peak is located between the satellites. Both scenarios result in an artificial transfer of spectral weight from the QP peak to the satellites. We observed the second scenario for every single 1s excitation in the CORE65 benchmark set^62^ because the optical gap (≈Ω^1^) is in the range of 5–15 eV and thus always smaller than |Δ_*n*_^QP^|.

We showed that including eigenvalue self-consistency in the propagator *G* solves this problem.^62,63^ In the eigenvalue self-consistent ev*GW*_0_ method, the DFT orbital energies in eq 11 are replaced with the current approximation for the QP energies ϵ_*m*_^QP^ in each iteration, while the excitations Ω^ν^ are kept at the RPA level.^39,62^ The correlation part of the ev*GW*_0_ self-energy is given by
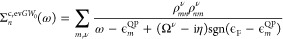
18The poles of Σ_*n*_^*c*, ev*GW*_0_^(ω) are at ϵ_*m*_^QP^ ± Ω^ν^, thus the satellites are placed relative to the QP energies ϵ_*m*_^QP^. This effectively restores the QP peak in core-levels, yielding absolute core-level binding energies within 0.3 eV of the experiment for ev*GW*_0_@PBE.^62,63^ The ev*GW*_0_ scheme comes at the price of increased computational cost compared to *G*_0_*W*_0_, since *all ϵ*_*m*_^QP^ need to be recalculated for every iteration of eq 17.

The ev*GW*_0_ scheme can be approximated by applying a global so-called “Hedin shift” Δ*H* to the self-energy, which is calculated once and is kept constant during the iterations of eq 17.^81^ For deep core-levels, we previously demonstrated that a level-specific shift Δ*H*_*n*_ instead of the global shift is required to approximate the ev*GW*_0_ solution adequately.^63^ Δ*H*_*n*_ is obtained from the first iteration of ev*GW*_0_ as
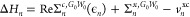
19The Hedin shift enters the correlation part of the self-energy Σ_*n*_^*c*, *G*_Δ*H*_*W*_0_^(ω) as
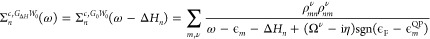
20The poles in Σ_*n*_^*c*, *G*_Δ*H*_*W*_0_^(ω), and consequently the satellites, are shifted by Δ*H*_*n*_ ≈ Δ_*n*_^QP^. We showed that Σ_1s_^*c*,*G*_Δ*H*_*W*_0_^ closely resembles Σ_1s_^*c*,ev*GW*_0_^ and that the 1s QP energies from *G*_Δ*H*_*W*_0_@PBE are comparable to those from ev*GW*_0_@PBE, with absolute core-level binding energies deviating by 0.25 eV from experiment.^63^

Using an approximate self-consistent scheme for *G* resolves the first problem discussed in Section 2.2: the poles in the self-energy are positioned relative to the QP energies rather than the DFT energies. However, the second and third problems remain. There are too few satellites, and they appear at more negative frequencies than the poles in the self-energy, with Δ^Sat–QP^ = Ω + |Δ^Pole^|. A consistently better description of the satellite region requires additional vertex corrections in *G*, such as those provided by *GW* + *C*.

## 3 *GW* + *C* for Molecules

### 3.1 Cumulant Expansion

In the following, we summarize the key equations of the *GW* + *C* approach, typically presented in the literature^44,45,48,82^ using a numerical scheme based on the spectral representation of the self-energy. We adopt this numerical scheme in our implementation (see Section 4.1). However, for this section, we use the analytical expressions derived in ref (60), employing eq 11, to facilitate the discussion on the validity of the method for shakeup satellites. The retarded Green’s function formalism is employed for the *GW* + *C* equations.^60,82^

The cumulant Green’s function *G*_*n*_^*C*^(*t*) for a hole takes the form^42,44^
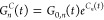
21where
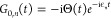
22is the retarded, time-dependent free propagator. Θ is the Heaviside function and *C*_*n*_ is the cumulant. The combination of eq 21 with the *G*_0_*W*_0_ self-energy yields the *G*_0_*W*_0_ + *C* approach, which we will refer to as *GW* + *C* for brevity.

We start by a Taylor expansion of the exponential ansatz given in eq 21
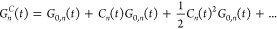
23Next, we assume that the cumulant is linear in *W*.^27,44,45^ An approximate expression for the cumulant *C*_*n*_(*t*) is derived by comparing eq 23 with the Dyson equation (eq 12) for the terms that are linear in the self-energy. Specifically, we compare the second term of eqs 23 and 13:
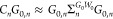
24Substituting eq 22 into eq 24, the time-dependent cumulant *C*_*n*_(*t*) can be linked to Σ_*n*_^*G*_0_*W*_0_^(ω) via a Fourier transform.
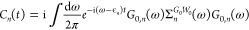
25We insert the retarded *G*_0,*n*_(ω), which corresponds to eq 2 replacing −iη sgn(ϵ_F_ – ϵ_*n*_) by iη, and shift the integration variable by ϵ_*n*_ to obtain
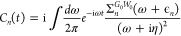
26

We separate the cumulant into a correlation *C*_*n*_^*c*^ and exchange *C*_*n*_^*x*^ contribution
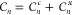
27*C*_*n*_^*x*^ is derived from the exchange part of the self-energy Σ_*n*_^*x*, *G*_0_*W*_0_^.
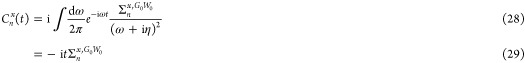
The correlation part *C*_*n*_^*c*^(*t*) is derived by inserting the retarded formulation of Σ_*n*_^*c*,*G*_0_*W*_0_^ (same as eq 11 replacing −iη sgn(ϵ_F_ – ϵ_*n*_) by iη)
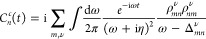
30where
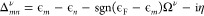
31We solve the integral in eq 30 analytically,^27,60^ resulting in the formulation known as the Landau form of the cumulant,^83^
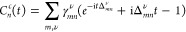
32where the excitation weights γ_*mn*_^ν^ are given by
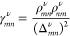
33The cumulant propagator is obtained by inserting eq 22 and eq 27 into eq 21
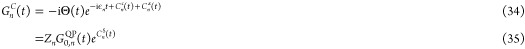
where we used eqs 32 and eq 29 to obtain eq 35. *Z*_*n*_ is a renormalization factor which originates from the time-independent term of *C*_*n*_^*c*^(*t*) (eq 32).
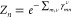
36*G*_0, *n*_^QP^(*t*) denotes the QP propagator and is given by
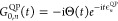
37
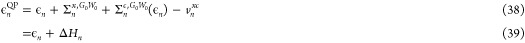
where Δ*H*_*n*_ corresponds to the level-specific Hedin shift defined in eq 19. ϵ_*n*_^QP^ denotes the QP energy at the *GW* + *C* level and corresponds to the *G*_Δ*H*_*W*_0_ QP energy (see Supporting Information (SI) Section S1). The *G*_0, *n*_^QP^(*t*) propagator arises from the sum of three terms: (i) the contribution – i*tϵ*_*n*_ from the free propagator *G*_*n*,0_(*t*) (eq 22), (ii) *C*_*n*_^*x*^ (eq 29) and (iii) the term linear in *t* in eq 32, for which we used the identity
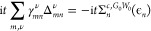
40Note that we must also subtract *v*_*n*_^*xc*^ in eq 38 when ϵ_*n*_ is a DFT eigenvalue. Furthermore, *C*_*n*_^S^(*t*) in eq 35 is given by
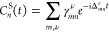
41which is derived from the exponential term in eq 32 and is the term which creates the satellites.

Next, we expand exp{*C*_*n*_^S^(*t*)} in a Taylor series
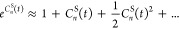
42Each individual order of *C*_*n*_^S^(*t*) gives rise to a series of features in the frequency-dependent propagator. Using eq 42, we obtain the Fourier transform of eq 35 as follows:
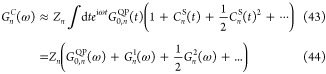
with
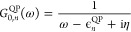
45
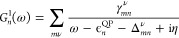
46
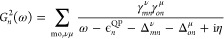
47The spectral function is given by
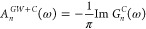
48which is slightly different compared to eq 15 because we formulated the *GW* + *C* approach in the retarded and not time-ordered formalism.

The poles in the real part of eq 44 correspond to peaks in *A*_*n*_^*GW*+*C*^. The analytic expanded form of *G*_*n*_^*C*^(ω) directly reveals where and what type of features are expected in the spectral function: *G*_0,*n*_^QP^, as defined in eq 45, generates the QP peak at ω = ϵ_*n*_^QP^. The first-order term, *G*_*n*_^1^(ω), eq 46, gives rise to satellite features at ω = ϵ_*m*_ – Ω^ν^ + Δ*H*_*n*_, corresponding to a single excitation Ω^ν^ coupling to the charged excitation. The second-order term introduces higher-order satellite features, where two uncorrelated charge-neutral excitations, Ω^ν^ and Ω^μ^, couple to the charged excitation. Triple and higher-order excitations are generated by the propagators *G*_*n*_^3,···^(ω). The intensities of the first, second, third, and higher-order satellites are distributed in a Poisson-like manner, as products of single-excitation intensities. Consequently, second and higher-order satellites only appear if strong single-excitations satellites are present.

The *GW* + *C* approach solves the shortcomings of *G*_0_*W*_0_ discussed in Section 2.2. First, for *n* = *m*, satellites now appear at positions relative to the QP peak, rather than relative to the DFT energies. Specifically, the first-order satellites associated with the level *n* are located at ω = ϵ_*n*_ – Ω^ν^ + Δ*H*_*n*_ = ϵ_*n*_^QP^ – Ω^ν^. Second, the poles at ω = ϵ_*n*_^QP^ – Ω^ν^ appear directly in the propagator *G*_*n*_^*C*^, which means that they directly show up in the spectral function *A*_*n*_^*GW*+*C*^, and we have Δ^Sat–QP^ = Ω^ν^. The third point, concerning the number of satellites, is discussed in Section 3.2.

### 3.2 Assessing the Validity of *GW* + *C* for a Shakeup Process in a Minimal Model

**Figure 2:**
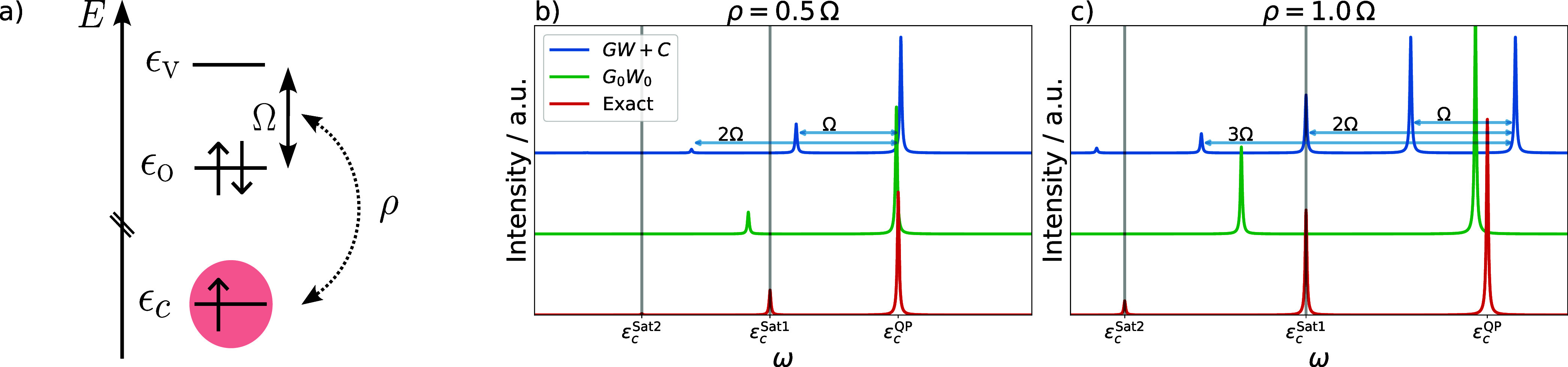
Three-level core-hole model system used by Cederbaum and co-workers in ref (13). Figure 2a sketches the molecular orbital diagram of the model, containing one occupied (ϵ_O_) and one virtual (ϵ_V_) valence level and an ionized core-level ϵ_*c*_. In the CVS approximation, the system has only a single excitation Ω coupled to the core level with a transition moment ρ. Figure 2b,c shows the *G*_0_*W*_0_ and *GW* + *C* spectral functions with various relative coupling strengths compared to the exact solution of the system.

The cumulant expansion is based on the exact solution of an infinite bosonic system.^42^ Collective bosonic excitations (plasmons) are predominantly found in solids, whereas Ω^ν^ rather represent Fermionic electron–hole pairs in the molecular case.^4^ For Fermions, the higher order terms *G*_*n*_^2, 3,···^(ω) contain nonphysical excitations which violate the Pauli principle. Here, we qualitatively discuss this fact for the three-level model system, sketched in Figure 2a. This model was already introduced by Cederbaum and co-workers^13^ in 1980. We refer the reader to ref (13) for an in-depth discussion of the exact and cumulant solutions.

In the model system, a single core hole is created in an orbital with an unperturbed energy ϵ_*c*_. The core hole couples to a valence single-particle excitation Ω from an occupied orbital with energy ϵ_*o*_ to a virtual orbital with energy ϵ_*v*_. Interactions of the valence space are not included and thus Ω = ϵ_*v*_ – ϵ_*o*_. The coupling is mediated by a transition moment ρ_*cc*_ = ρ. The transition moments mixing core- and valence levels, i.e., ρ_*co*_ and ρ_*cv*_, are neglected following the core–valence separation (CVS) approximation.^11,13^ In the same spirit, charge-neutral core–valence excitations from *c* to *v* are omitted. The exact, *GW* + *C* and *G*_0_*W*_0_ spectral functions of this model system are shown in Figure 2b and c for two different ratios of ρ/Ω (0.5 and 1.0). The ratio ρ/Ω determines the effective coupling strength between the core hole and the excited state in all three approaches. For *GW* + *C*, this relationship is directly evident from eq 33, where the excitation weight for the model system is given by γ = ρ^2^/Ω^2^.

Starting with the exact solution, we expect two satellites in addition to the QP peak, ϵ_*c*_^QP^: the first satellite at ϵ_*c*_^Sat1^ corresponds to a single excitation where one electron is promoted from state *o* to *v*. The second, ϵ_*c*_^Sat2^, corresponds to a double excitation where both electrons in *o* are excited to *v*. The intensity of the second satellite is significantly smaller because it couples only indirectly with the ground state configuration. Triple- and higher order excitations are not possible, as the model considers only valence shell excitations and the valence shell contains only two electrons. The positions and intensities of the satellites depend on the ratio ρ/Ω. Increasing ratios enhance the intensities of the satellites and increase the separation between QP peak and satellite positions, see Figure 2b and c.

In the *GW* + *C* approximation, each order of *G*_*n*_^1, 2, 3,···^(ω) creates exactly one satellite peak because there is only one excitation weight γ. The satellites occur at ϵ_*c*_^QP^ – *n*Ω where , and the intensities follow the Poisson distribution. The intensity of each satellite is proportional to γ^*n*^, thus intensities depend on the ratio ρ/Ω, as for the exact solution. For ρ/Ω = 0.5, only the first two satellites (corresponding to single and double excitations) carry considerable spectral weight, whereas four satellites with nonvanishing intensity are visible for ρ/Ω = 1.0. The third and fourth satellite correspond to triple and quadruple excitations, which violate the Pauli principle because we can have only two electrons in state *v*, and only two electrons can be excited from *o*. In addition to this qualitatively wrong description, we find that the positions of the first two satellites deviates strongly from the exact solution for ρ/Ω = 1.0, whereas the agreement is better for ρ/Ω = 0.5.

In *G*_0_*W*_0_ only a single satellite is generated, regardless of the coupling strength. The satellite appears at higher binding energies compared to the exact and *GW* + *C* solution, consistent with the discussion in Section 2.2. We emphasize that for this minimal model with only a single higher-order excitation, the error of *G*_0_*W*_0_ and *GW* + *C* is of comparable magnitude. For realistic systems with many electrons and a large virtual space, we expect an improvement of *GW* + *C*, since the number of physical higher-order excitations captured by *GW* + *C* grows rapidly with the system size.

To avoid spurious higher-order satellites, one could simply neglect the terms *G*_*n*_^2,3···^, as done in ref (60). However, a separation into lower-order terms *G*_*n*_^1^ and higher order terms *G*_*n*_^2,3···^ as in eq 44 is not possible in our numerical implementation for large systems described in Section 4.1. Instead, we derive conditions under which a cumulant approach like *GW* + *C* is approximately valid, following the discussion of Cederbaum and co-workers in ref (13). For the model system, they formulated the following inequality
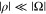
49If the inequality is kept, the excitation weights γ are small and the intensity of higher order (nonphysical) contributions quickly vanishes due to the Poisson distribution. This corresponds rather to a case as shown in Figure 2b.

In cases where eq 49 is violated, spurious contributions from higher-order terms must be eliminated. This can be achieved by employing nonlinear variants of the cumulant expansion instead of the linear parametrization in eq 24.^84,85^ Different paths like connections to real-time time-dependent DFT^85^ and real-time coupled cluster approximations^135−28^ have been proposed and applied to model systems or small molecules. The combination of *GW* with a nonlinear cumulant approach is has not been achieved yet and is also not the goal of the present work. Here, we aim to demonstrate that *GW* + *C* provides reliable results for molecular core-level satellites generalizing the inequality (eq 49) for the many-electron case.

### 3.3 Assessing the Validity of *GW + C* for Molecular Shakeups

For a realistic system with many orbitals, first order satellites in *GW* + *C* appear at frequencies
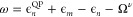
50in accordance with eq 46. The inequality in eq 49 is then generalized to
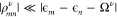
51

Equations 50 and 51 cover two different types of satellites in the spectral function of a level *n*: Satellites generated by the diagonal elements (*m* = *n*) and satellites generated by the off-diagonal elements (*m* ≠ *n*). For the first case, the DFT orbital energies in eq 50 vanish, and the satellites are placed relative to the QP peak at frequencies ω = ϵ_*n*_^QP^ – Ω^ν^. These satellites correspond to shakeup states, i.e., charge neutral excitations coupled to the ionized (core) level *n*. We will show this explicitly in Section 6.1.

We provide an example demonstrating that the inequality (eq 51) is satisfied for the shakeup satellites. Table 1 lists the transition energy, Ω^Sat1^, and the corresponding transition moments, ρ_C1s,m_^Sat1^, of the lowest symmetry-allowed satellite in the C 1s spectral function of an isolated CH_4_ molecule, calculated using different Gaussian basis sets. For the diagonal elements eq 51 simplifies to |ρ_C1s,C1s_^ν^| ≪ |Ω^ν^|. For the CH_4_ example, this inequality is clearly satisfied, as ρ_C1s,C1s_^Sat1^ is two orders of magnitude smaller than Ω^Sat1^ across all basis sets. Since molecules have typically large optical gaps of several electronvolts, it is reasonable to assume that eq 51 is generally holds for molecular 1s levels.

**Table 1:** Transition Moments ρ_*nm*_^Sat1^ and Excitation Energy Ω^Sat1^ of the Lowest Shakeup Satellite with a Non-Zero Coupling in CH_4_ Computed with the Fully Analytical *G*_0_*W*_0_ Implementation in PySCF^86^ a

basis set	Ω^Sat1^	ρ_C1s,C1s_^Sat1^	ρ_C1s,2_^Sat1^	ρ_C1s,3=4=5_^Sat1^
cc-pVTZ	13.91	0.3352	5.366 × 10^–7^	0.0000
cc-pVQZ	13.22	0.2099	9.258 × 10^–7^	0.0000
cc-pV5Z	12.58	0.2453	6.413 × 10^–7^	0.0000

a All values in eV.

Satellites due to the off-diagonal elements appear at frequencies ω = ϵ_*m*_ – Ω^ν^ + Δ*H*_*n*_. These satellites, previously termed *correlation satellites*,^10^ couple the ionization of a level with energy ϵ_*m*_ to the core orbital *n*. Or in other words, a shakeup peak in the spectral function of a level *m*, for example the highest occupied molecular orbital (HOMO), shows also up as correlation satellite in the spectral function of the core-level *n*. Generally, we expect the intensity of correlation satellites to be very small because the off-diagonal transition moments ρ_*mn*_^ν^ are negligible. This expectation is supported by the data in Table 1, where ρ_C1s,*m*_^Sat1^ is found to be orders of magnitude smaller than ρ_C1s,C1s_^Sat1^ for the semicore state (*m* = 2) and vanishes entirely for the valence states (*m* = 3–5).

Although the transition moments ρ_C1s,*m*_^ν^ are very small, eq 51 may still be violated because the right-hand side can be zero by chance if ϵ_*m*_ – ϵ_*n*_ = Ω^ν^. If *m* is a valence state and *n* a 1s core-level this implies that Ω^ν^ > 250 eV because, for example, C 1s, N 1s, and O 1s core-levels are around −290, −410, or −540 eV respectively, while valence excitations are typically in the order of tens of eV. Such large RPA excitation energies Ω^ν^ are mostly due to transitions from valence states to very high lying virtual states. In a plane-wave basis-set framework, these high lying virtual states are correctly described as continuum, and transitions to these states should appear as smooth offset in the spectral function. However, using a localized basis set, which is a computationally efficient choice for all-electron core-level calculations, the high-level virtual states are discrete,^87^ which we will also demonstrate in Section 6.1. The valence transitions to the high-lying states then generates sharp peaks in the spectral functions, which might gain weight if eq 51 is not satisfied.

### 3.4 Core–Valence Decoupling

The spurious contributions from correlation satellites can be removed by making use of the *decoupling approximation* for core-levels *c* in eq 11.^45^
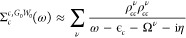
52In the decoupling approximation, only the contributions of core-level shakeup satellites are kept in the self-energy. The approximation is well justified for deep core-levels, since density shifts to other levels are very small, i.e., ρ_*cm*_^ν^ ≈ 0 (see Table 1). The situation is slightly more complicated in the case of a core-level delocalized over several *N*_Sym_ symmetry-equivalent cores, e.g., in benzene. In that case, all contributions from symmetry-related levels have to be considered.
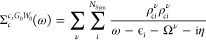
53Such a drastic simplification as the decoupling approximation comes at a price, specifically the QP shift is altered due to the missing relaxing valence orbital contributions. However, we can calculate the satellite term *C*_*n*_^S^ independently of the QP peak *G*_0,*n*_^QP^, applying the decoupling approximation only to the satellite spectrum.

The decoupling approximation follows a similar spirit as the CVS approximation, which is applied in many different flavors of wave function methods, e.g., in ADC^13,88−90^ or CC^91,92^ approaches, and exploits the low overlap between core- and valence region by systematically neglecting integrals involving products of core- and valence orbitals.^11^ However, both approximations are not exactly equal, since we do not apply a similar approximation for the calculation of the RPA couplings and include integrals of the type (*cc*|*ca*) in ρ_*cc*_^ν^, which would be zero in the CVS approximation.

## 4 Implementation

### 4.1 Numerical *GW* + *C* Scheme

In our implementation, the working equations do not rely on the fully analytical expressions given in Section 3.1. Instead, we employ the more commonly used numerical *GW* + *C* scheme,^44,45,52,81,82^ widely adopted within the solid-state physics community. In this numerical *GW* + *C* approach, we employ the spectral representation of the self-energy.^45,82,93,94^
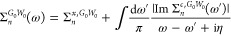
54We obtain the cumulant *C*_*n*_(*t*) by inserting eq 54 into eq 26. The exchange term *C*_*n*_^*x*^(*t*) is given by eq 29 as before. The correlation part *C*_*n*_^*c*^(*t*) is obtained from a double-frequency integral
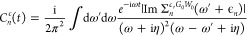
55Carrying out the integration over ω and relabeling ω′as ω gives the Landau form of the cumulant, in analogy to eq 32.
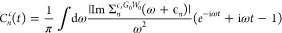
56We split *C*_*n*_^*c*^(*t*) into
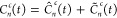
57where
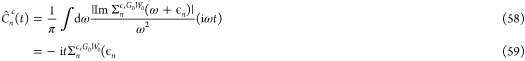
and
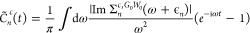
60The cumulant Green’s function *G*_*n*_^*C*^(*t*) is derived by inserting eq 22 and eq 27 into eq 21, using eqs 57 and 29 for *C*_*n*_^*c*^(*t*) and *C*_*n*_^*x*^, respectively. Furthermore, we use eq 59 for *Ĉ*_*n*_^*c*^(*t*) and absorb this contribution into the QP propagator *G*_0,*n*_^QP^ defined in eq 37, yielding
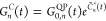
61In the numerical scheme, the renormalization and satellite terms, *Z*_*n*_ and exp{*C*_*n*_^S^(*t*)}, are contained in exp {*C̃*_*n*_^*c*^(*t*)}.

The frequency-dependent propagator is computed by making use of the convolution theorem.
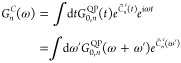
62*G*_0, *n*_^QP^(ω) is known from eq 45, whereas *e*^*C̃*_*n*_^*c*^(ω)^ has to be computed numerically from the Fourier transform.
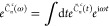
63Our final quantity, the spectral function *A*_*n*_^*GW*+*C*^(ω), is obtained from *G*_*n*_^*C*^(ω) as in eq 48.

*A*_*n*_^*GW*+*C*^ can be approximated in terms of Im Σ_*n*_^*c*,*G*_Δ*H*_*W*_0_^(ω) = Im Σ_*n*_^*c*,*G*_0_*W*_0_^(ω – Δ*H*_*n*_) by making use of a Taylor expansion. To first order, the spectral function is approximated as
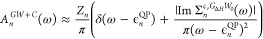
64A detailed derivation of eq 64 is provided in Section S2 in the SI. Following eq 64, every peak in ImΣ_*n*_^*c*,*G*_Δ*H*_*W*_0_^ is directly related to a satellite peak in *A*_*n*_^*GW*+*C*^(ω).

### 4.2 Workflow

We implemented the *GW* + *C* approach in the FHI-aims package.^95^ FHI-aims is an all-electron electronic structure code based on numeric atom-centered orbitals (NAOs) defined as
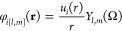
65The angular dependency is captured by the complex spherical harmonics *Y*_*l*,*m*_, while the radial part *u*_*i*_ is evaluated on a numerical grid and thus very flexible. Standard quantum chemistry basis sets, e.g. Gaussian type orbitals (GTOs) or Slater type orbitals (STOs), are a special case of eq 65 where *u*_*i*_(*r*) is a Gaussian or Slater function.

**Figure 3:**
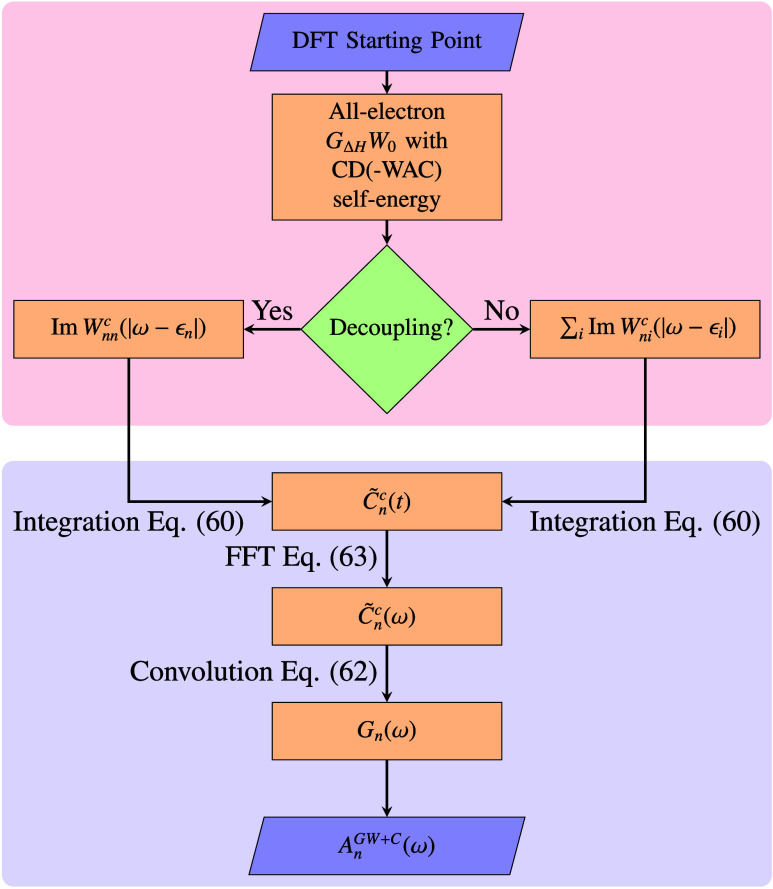
Flowchart of the *GW* + *C* workflow. The red-shaded part comprises a standard *G*_Δ*H*_*W*_0_ calculation as implemented in the FHI-aims package. Subsequently, the newly implemented *GW* + *C* procedure (blue-shaded) is carried out as a postprocessing step.

Equations 60, 62, and 63 were implemented as postprocessing step following a *GW* calculation. The workflow of our implementation is summarized in Figure 3. For convenience, we use the *G*_Δ*H*_*W*_0_ scheme instead of *G*_0_*W*_0_ as the starting point for the *GW* + *C* calculation, as it directly provides the *GW* + *C* QP energy ϵ_*n*_^QP^ defined in eq 38, which is needed to calculate *G*_*n*,0_^QP^. We also avoid numerical artifacts, such as the nonconvergence of the QP eq 17 at the *G*_0_*W*_0_@PBE level, due to the multisolution behavior for core-levels discussed in Section 2.3. In the *GW* step, Im Σ_*n*_^*c*,*G*_Δ*H*_*W*_0_^(ω) is evaluated on a discrete, equidistant frequency grid using the CD or CD-WAC technique. If the decoupling approximation eq 52 is invoked, Σ_*n*_^*c*,*G*_Δ*H*_*W*_0_^(ω) contains only the diagonal part of the screened interaction, as discussed in more detail ins Section 4.3.

Next, we evaluate *C̃*_*n*_^*c*^(*t*), using the Hedin-shifted self-energy matrix elements as input following eq 20. The frequency integral in eq 60 is carried out numerically for a set of *n*_*t*_ discrete times, with time steps chosen such that we obtain the desired energy resolution after the final Fourier transform. Due to the smooth nature of the imaginary part of the self-energy, optimized integration grids, which are commonly applied in *GW*,^96,97^ are not necessary. The Fourier transform from time-to-frequency space in eq 63 is performed using the fast Fourier transform (FFT) utility of the FFTW package for a set of *n*_FFT_ frequencies.^98^ Finally, we obtain the propagator in eq 62 in the desired frequency range by numerically integrating over the FFT frequencies ω′.

The computational cost is dominated by the evaluation of the self-energy matrix elements Σ_*n*_^*c*,*G*_Δ*H*_*W*_0_^(ω). The contribution of the cumulant evaluation to the total computational time is negligible. While the analytical *GW* + *C* formulation in Section 3.1 requires an analytic evaluation of the self-energy, resulting in an unfavorable *O*(*N*^6^) scaling with system size *N*, our numerical implementation leverages the lower-scaling CD and CD-WAC algorithms for evaluating Σ_*n*_^*c*,*G*_0_*W*_0_^(ω).

### 4.3 Self-Energy Evaluation

For core-level calculations, we use the CD technique to perform the frequency integration of the self-energy. The CD implementation in FHI-aims is based on the resolution-of-the-identity (RI) approximation^99,100^ and is described in detail in our previous publication.^61^ The CD technique allows the numerical exact evaluation of the self-energy at a given real frequency.

In the CD formulation, the integral eq 4 for the correlation part is divided into two parts, one including an integral over the imaginary axis *I*_*n*_(ω) and a residue part *R*_*n*_(ω), which sums up the residues in *G*_0_.
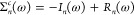
66The integral along the imaginary axis is defined as
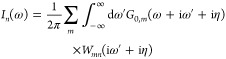
67and the residue term is given by
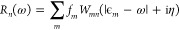
68The prefactors *f*_*m*_ are defined as
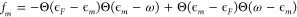
69For a frequency in the core-level range, the sum in eq 68 runs over all occupied levels. Since the calculation of *W*_*mn*_ at a specific frequency scales *O*(*N*^4^) with respect to system size *N*, this leads to an overall scaling of *O*(*N*^5^) for *R*_*n*_. The evaluation of *I*_*n*_ is *O*(*N*^4^); for a detailed discussion of the scaling we refer the reader to ref (61).

The central idea of the CD-WAC approach^72,76^ is to reduce the scaling of the *R*_*n*_ term to *N*^4^ by approximating the screened interaction *W* using analytical continuation (AC) techniques, specifically a Padé approximation eq 70.
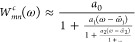
70To obtain the parameters *a*_*i*_, we compute the matrix elements *W*_*mn*_^*c*^(ω̃) for a fixed number of reference frequencies ω̃. The reference frequencies ω̃ are taken along the real- and the imaginary axis. The points along the imaginary axis are reused from the numerical integration in eq 67. The real-valued frequencies are picked in the core/valence region, and the range is determined by the numerical parameters ω_C_^min/max^ for the core region and Δ_lower/upper_ for the valence region. The number of real-frequency reference points in the core- and valence region (*N*_C_^WAC^/*N*_V_^WAC^) determines the accuracy of the fit. Sensible choices for these parameters were derived in ref (76).

After the Padé fit, the computation of *W*_*mn*_(ω) requires only the evaluation of an analytic function, reducing the overall scaling to *N*^4^ for core-levels. In ref (76), we demonstrated the scalability of the CD-WAC implementation, achieving a 10-fold speed-up in the QP calculations for the largest system, consisting of 116 atoms, compared to CD. The reduction in computational cost is particularly significant when calculating spectral functions because this requires evaluating the self-energy at several thousand frequency points, compared to only 10–15 points when iterating the QP eq 17. The CD-WAC speed-up with respect to CD is in the range of a factor of 100 to 1000, depending on the chosen frequency range and resolution of *A*_*n*_^*GW*+*C*^(ω). Practically, CD-WAC produces spectral functions with negligible computational overhead after the QP evaluation.

In CD(-WAC), the integral in *I*_*n*_(ω) is real for η → 0 due to the symmetry relation *W*_*mn*_(iω) = *W*_mn_^*^(−iω).^94^ Therefore, the imaginary part of the self-energy, which is required for the calculation of the cumulant in eq 60, depends only on *R*_*n*_(ω), and we can rewrite Im Σ_*n*_^*c*,*G*_0_*W*_0_^(ω) as
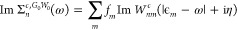
71By inspecting eqs 5 and 52, we note that the decoupling approximation eq 52 can be enforced by approximating
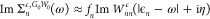
72For symmetry related core-levels, all contributions from equivalent core-levels have to be included as in eq 53.

## 5 Computational Details

We performed all-electron *G*_Δ*H*_*W*_0_ and *GW* + *C* calculations for the CORE65 benchmark set^62^ as well as for the acene series *C*_4*n*+2_*H*_2*n*+4_ (*n* = 1–5) using the FHI-aims program package.^95,99^ The CORE65 benchmark set includes 65 1s core-level binding energies for 32 organic molecules containing the elements H, C, N, O, and F, consisting of one open-shell and 31 closed-shell systems.^62^ The geometries for the benchmark set are available in the original publication.^62^ We generated the acene structures for *n* > 1 by performing geometry optimizations at the DFT level using NAOs of *tier 2* quality (FHI-aims-2020 default). We employed the PBE exchange–correlation functional^78^ including van-der Waals interactions via the Tkatchenko-Scheffler dispersion correction^101^ and scalar-relativistic effects using the zeroth order regular approximation (ZORA).^95^

We used the PBE functional as starting point for the nonrelativistic *G*_Δ*H*_*W*_0_ calculations and the CD(-WAC) technique for the frequency integration. The integration along the imaginary axis (eq 67) was performed using a 200-points modified Gauss-Legendre grid. For the CD-WAC calculation, we calculated *W*_*mn*_^*c*^ at 400 reference frequency points, consisting of 200 imaginary frequencies from the modified Gauss-Legendre grid and another 200 real-valued frequencies points, with *N*_C_^WAC^ = *N*_V_^WAC^ = 100. The frequency ranges for the real points are ω_C_^min^ = 0 eV, ω_C_^max^ = 40 eV for the core region and Δ_lower_ = 8%, Δ_upper_ = 1% for the valence region (see ref (76)). To account for relativistic effects in the absolute QP energies, we applied the scalar-relativistic corrections derived in ref (71) (see Section S5).

We used several common Gaussian- and NAO-type all-electron basis sets for convergence studies: The Gaussian basis sets comprise Dunning’s correlation-consistent basis sets (cc-pV*X*Z,^102^*X* = 3–6), with additional diffuse functions (aug-cc-pV*X*Z,^103^*X* = 3–6) and with core-optimized (C) functions (aug-cc-pCV*X*Z, *X* = 3–6) as well as the core-rich basis sets of the ccX-*X*Z family (*X* = 3–5).^67,104^ For the NAO basis sets, we employed the FHI-aims-2020 *tier 2* (T2) basis set, augmented with two additional diffuse (+aug2) functions obtained from the (*l* = 0, 1) augmentation functions of the aug-cc-pV5Z basis set.^105^ Furthermore, a set of highly localized Slater functions (STO*X*) was added to the NAO basis sets, abbreviated as T2+aug2+STO*X* (*X* = 1–5).^106^

As a measure for the basis set convergence, we assess the splitting Δ^Sat1–QP^ of the lowest nonvanishing satellite at ϵ^Sat1^ and the QP peak.
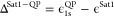
73For every basis set, we calculated the absolute error with respect to the largest basis set utilized here (aug-cc-pCV6Z) and averaged over all 65 1s core-levels in the CORE65 benchmark set to obtain to the mean average error (MAE):
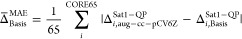
74The position of the lowest satellite is determined based on a intensity criterion outlined in Section S3 in the SI.

For *GW* + *C* calculations, we used *n*_t_ = *n*_FFT_ = 2^18^ and a final energy resolution of 0.01 eV. An imaginary broadening parameter η = 0.1 eV is used throughout the *GW* + *C* calculations if not stated otherwise. The input and output files of all the FHI-aims calculations are available in the NOMAD database.^107^

Reference RPA, time-dependent DFT (TDDFT) and analytical transition moments calculations were carried out in PySCF.^86,108,109^

## 6 Results and Discussion

We start with assessing the validity of the decoupling approximation, introduced and motivated in Section 3.4, and proceed with the analysis of important technical settings like the frequency integration technique, the basis set dependence and the choice of the DFT starting point. We also compare the *GW* + *C* spectral functions of small molecules with both experimental data and *G*_Δ*H*_*W*_0_ spectral functions. Finally, we showcase a possible application of our approach for the interpretation of experimental data by studying the acene series from benzene to pentacene.

### 6.1 Decoupling Approximation

**Figure 4:**
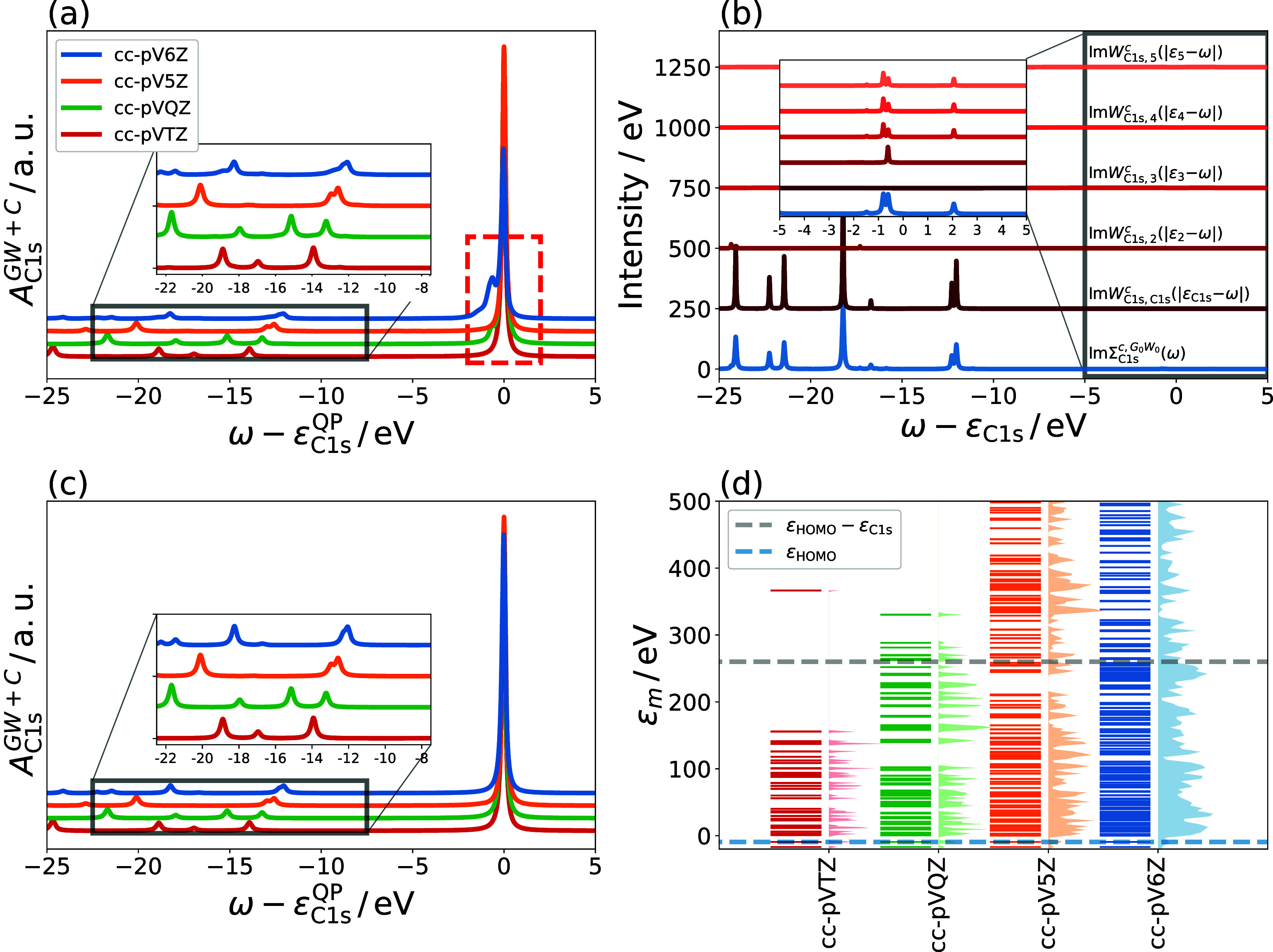
Analysis of the decoupling approximation for the C 1s core-level in CH_4_. (a): *GW* + *C* spectral function of the satellite region using the full self-energy as in eq 71. (b): Contributions to ImΣ_C1s_^*c*,*G*_0_*W*_0_^(ω) from diagonal- and off-diagonal elements of the screened interaction in the cc-pV6Z basis. Low-intensity features close to the QP peak are highlighted in the inset, demonstrating that such spurious features arise from the off-diagonal elements of the screened interaction. In the *GW* + *C* spectral function, these satellites gain intensity following eq 51. (c): Spectral functions obtained after applying the decoupling approximation (eq 72), yielding a satellite spectrum free of spurious correlation satellites. (d): Energy distribution of the DFT orbital energies ϵ_*m*_ for the occupied valence states and virtual states, showing their exact positions as horizontal lines and their Gaussian-broadened distribution as a shaded plot.

In Figure 4a, the spectral function *A*_C1s_^GW+C^ of CH_4_ is displayed for the cc-pV*X*Z (*X* = 3–6) basis set family without using the decoupling approximation. For convenience, we center the spectral functions at the C 1s QP peak. For all basis sets, we observe the occurrence of a series of shakeup satellites between −12 and −20 eV, as shown in the inset. However, when employing the cc-pVQZ and cc-pV6Z basis sets, additional signals appear near the QP peak, highlighted by the red dashed box. These peaks carry considerable spectral weight, but have no counterpart in the cc-pV5Z and cc-pVTZ basis sets. For the cc-pV6Z basis set, a very intense peak appears roughly −1 eV from the QP peak, at least 1 order of magnitude more intense compared to the satellites in the inset. Due to the multiplicative nature of the cumulant eq 42, this peak creates a set of replicas, i.e., higher order satellites, on top of every peak in the spectrum, causing additional broadening and peaks in the spectrum.

To analyze the origin of the satellites close to the QP peak (red dashed box in Figure 4a), we first note that satellites in *A*_*n*_^*GW*+*C*^ can be either shakeup satellites or correlation satellites, as discussed in Section 3.3. Shakeup satellites appearing at *A*_*n*_^*GW*+*C*^(ω ≈ ϵ_*n*_^QP^) require a very small charge-neutral excitation energy of Ω^ν^ ≈ 0, while correlation satellites coupling valence- and core-levels need to have very large excitation energies of Ω^ν^ > 250 eV to show up in the C 1s core region. For the satellite in the cc-pV6Z spectral function at ω ≈ ϵ_C1s_^QP^, a shakeup satellite would require an RPA excitation with Ω^ν^ ≈ 1 eV. For CH_4_, such an excitation does not exist, since the lowest RPA excitation energy in CH_4_ is above 10 eV. In turn, a correlation satellite, coupling e.g., HOMO and C 1s level, requires an RPA excitation energy of roughly Ω^ν^ ≈ 260 eV to appear at the same frequency in the spectral function, i.e., at ω = ϵ_C1s_^QP^ + ϵ_HOMO_ – ϵ_C1s_– Ω^ν^ ≈ ϵ_C1s_^QP^ (eqs 31 and 46), where ϵ_C1s_^QP^ = −290 eV, ϵ_HOMO_ = −9.4 eV and ϵ_C1s_ = −268.5 eV for that particular example.

To assess the likelihood of such high-energy RPA excitations, we display the energy distribution of the virtual states with increasing basis set size in Figure 4d. The RPA excitations Ω^ν^ are close to the orbital differences ϵ_*a*_ – ϵ_*i*_, and therefore the DFT eigenvalues can indicate the occurrence of RPA excitations with matching energy. Near the Fermi level (ϵ_*m*_ ≈ 0 – 20 eV), the number of virtual states increases rapidly with the basis set size, and the number of high-energy states extending to several hundred electronvolts also grows from cc-pVTZ to cc-pV6Z. However, the density of states at high energies does not become continuous; instead, discrete levels with large gaps between them are observed.

For the small cc-pVTZ basis set, there are no states in the 250–300 eV range. Consequently, Ω^ν^ around 260 eV do not exist and only a pure shakeup spectrum is observed in Figure 4a. In contrast, for the cc-pVQZ, cc-pV5Z, and cc-pV6Z basis sets, states are present in the 250–300 eV range, and correlation satellites do appear in the spectral function, as shown in Figure 4a. The number of states in this energy range increases with larger basis sets, making the occurrence of correlation satellites more likely for the largest basis set.

For a deeper analysis and to justify the decoupling approximation, we trace the correlation satellite contributions in our numerical *GW* + *C* scheme by plotting Im Σ_C1s_^*c*^(ω) for the cc-pV6Z basis set in Figure 4b. Following eq 64, the peaks in Im Σ_*n*_^*c*^(ω) directly show up with modulated intensity in the *GW* + *C* spectral function as satellites. Furthermore, we plot the individual contributions to Im Σ_*n*_^*c*^(ω) in Figure 4b, i.e., the matrix elements Im *W*_C1s,*m*_^*c*^(|ϵ_*m*_ – ω|) (see eq 71). Methane has five occupied states and we have consequently five contributions because the sum over *m* in eq 71 includes all occupied levels for the frequency range under consideration (ω < ϵ_C1s_).

In Figure 4b, the most intense peaks in Im Σ_C1s_^*c*^ appear in the region between −25 to −12 eV, originating solely from the diagonal element Im *W*_C1s,C1s_^*c*^(|ϵ_C1s_ – ω|). For ω < ϵ_C1s_, peaks in Im *W*_C1s,C1s_^*c*^(|ϵ_C1s_ – ω|) are due to Ω^ν^ = 12–25 eV, as evident from eq 5 (poles in the real part correspond to peaks in the imaginary part). These are transitions from valence states to low-energy virtual states, generating the actual shakeup satellites. In addition, we observe small peaks for the off-diagonal elements Im *W*_C1s,2–5_^*c*^(|ϵ_2–5_ – ω|) between −5 to 5 eV, see inset in Figure 4b. Following eq 5, these peaks correspond to Ω^ν^ > 250 eV because ϵ_2–5_ ranges from −17 to −9.4 eV. This demonstrates that the off-diagonal elements Im *W*_C1s,2–5_^*c*^(|ϵ_2–5_ – ω|) are responsible for the correlation satellites. However, the intensity of the off-diagonal contributions is orders of magnitude smaller compared to those from the diagonal elements. Nevertheless, the off-diagonal contributions in Im Σ_C1s_^*c*^ gain artificial spectral weight in *GW* + *C* because they appear very close to the QP peak. This follows directly from inspecting the second term in eq 64, corresponding to violation of the inequality (eq 51).

The correlation satellites appear at random positions and are likely to occur in large basis sets, which are needed to converge the calculations, see Section 6.3. Therefore, it is crucial to remove spurious correlation satellites from Im Σ_C1s_^*c*^ to prevent a breakdown of the *GW* + *C* approximation. To achieve this, we apply the decoupling approximation, as outlined in Section 3.4. Following eq 72, we neglect all off-diagonal elements Im *W*_C1s,2–5_^*c*^(|ϵ_2–5_ – ω|), thereby effectively removing all correlation satellites. As demonstrated in Figure 4c, the resulting spectrum is ”clean” and the shakeup spectrum is equally well captured as in Figure 4a.

### 6.2 CD-WAC

We showed in ref (76) that CD-WAC reproduces QP energies within 4 meV of the CD reference. Here, we briefly comment on the accuracy of the CD-WAC approximation for spectral functions.

**Figure 5:**
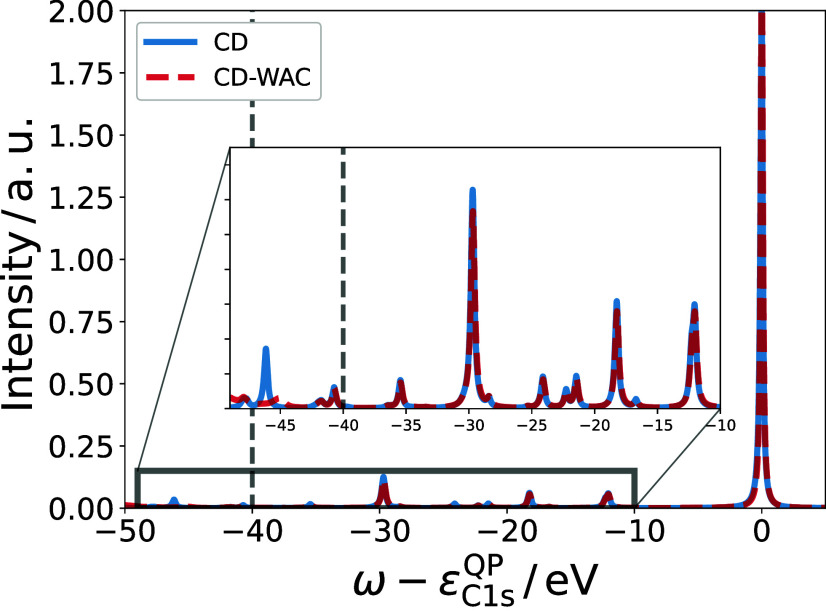
Comparison of *A*_C1s_^*GW*+*C*^ of CH_4_ (cc-pV6Z basis, with decoupling approximation) computed with CD and CD-WAC self-energies. The fitting threshold ω_C_^max^ is indicated by a gray line.

In Figure 5, the *GW* + *C* spectral functions based on CD- and CD-WAC self-energies are compared for CH_4_, employing the decoupling approximation. Both *GW* + *C* spectral functions match well, with satellite positions and intensities in excellent agreement to each other. Beyond the fitting threshold ω_C_^max^ (i.e., more than 40 eV from the QP peak, as indicated by the dashed gray line), the accuracy of the CD-WAC fit begins to deteriorate, and the satellites are slightly shifted relative to the CD reference. In this work, we are primarily interested in low-lying satellites within a few eV of the QP peak, which is accurately described by the CD-WAC fit. Moreover, the satellites beyond −40 eV of the QP energy are mostly excitations to the continuum, which appear as spurious peaks in a localized basis set as discussed in Section 3.3.

We note that CD-WAC fits also the off-diagonal elements *W*_C1s,*m*_^*c*^ well and reproduces therefore also the correlation satellites (see Section S4 in SI). Consequently, the decoupling approximation must be also employed for *GW* + *C* calculations based on a CD-WAC self-energy.

### 6.3 Basis Set Convergence

We investigate the basis set dependence of the satellite spectrum using the CORE65 benchmark set, employing four different GTO basis set families and customized NAO basis sets as detailed in Section 5.

**Figure 6:**
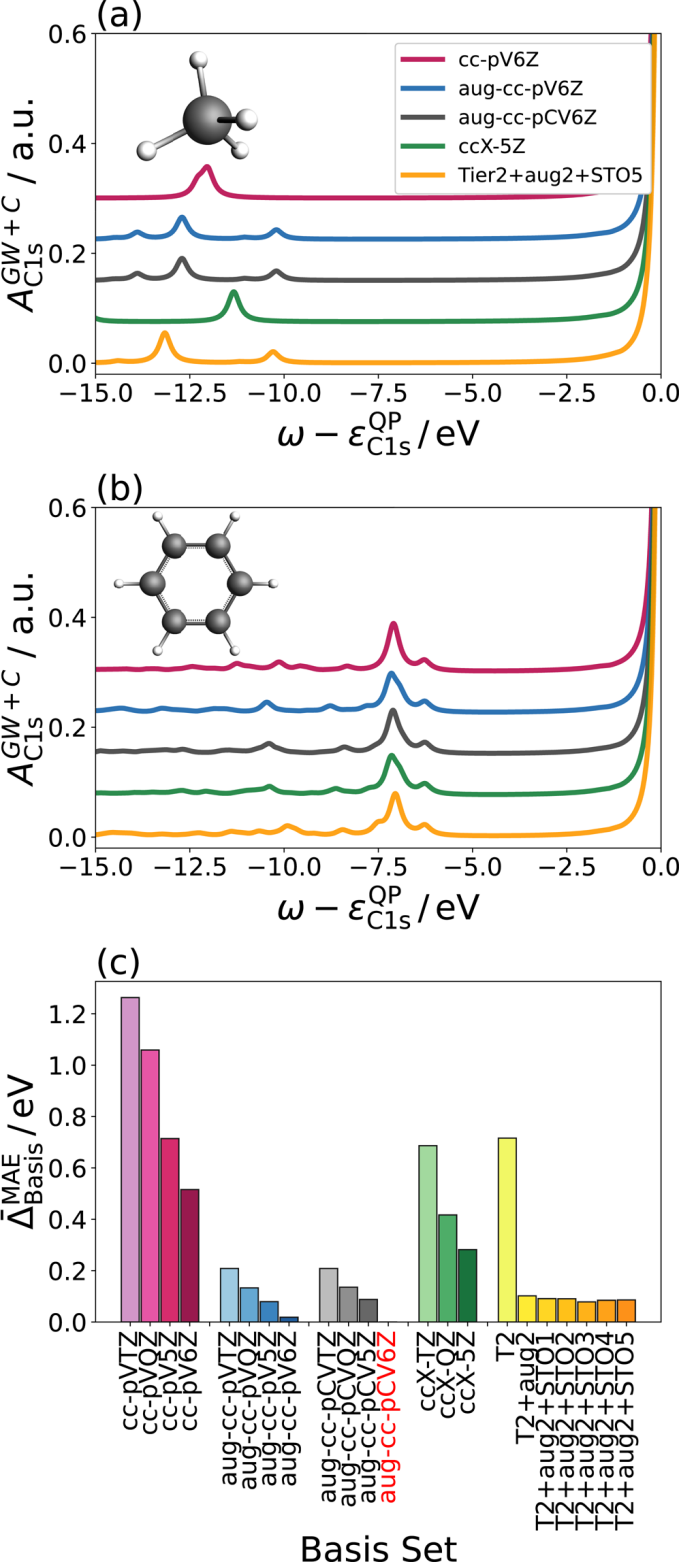
Basis set convergence of the lowest-energy satellite for the CORE65 benchmark set relative to the largest basis set (aug-cc-pCV6Z, marked in red). Panels (a, b) show the satellite spectrum of the CH_4_ and C_6_H_6_ C1s level for the largest basis set of each family. Panel (c) displays Δ̅_Basis_^MAE^ relative to the aug-cc-pCV6Z basis set as specified in eq 74.

The effect of different basis sets is displayed in Figure 6a and b for methane (CH_4_) and benzene (C_6_H_6_) using the largest basis set of each family. For C_6_H_6_ in Figure 6b, all basis sets yield nearly identical satellite spectra, with the onset of the satellite region at −6 eV and a dominating peak at −7 eV. For CH_4_ in Figure 6a, the basis sets with diffuse (aug-) functions produce similar spectra, with a first satellite appearing at ca. −10 eV and a second, more intense satellite around −13 eV. However, the cc-pV6Z and ccX-5Z spectra lack the peak near −10 eV and instead show more intense satellite peaks around −12 and −11 eV. This suggests that the basis set dependence varies significantly across different systems.

For a more systematic assessment, we computed *A*_1*s*_^*GW*+*C*^ for the 65 excitations in the CORE65 benchmark set and calculated Δ^Sat1–QP^ (eq 73) for each spectral function. The MAE of Δ^Sat1–QP^ with respect to the aug-cc-pCV6Z result, see definition in eq 74, is shown in Figure 6 for all 22 basis sets. For completeness, we report the MAEs of the 1s QP excitations with respect to experiment and with respect to the aug-cc-pCV6Z result for each basis set in Section S5 of the SI.

In Figure 6c, we observe that for the correlation-consistent (cc) Gaussian basis set families, Δ̅_Basis_^MAE^ systematically decreases with increasing basis set size, reducing the error by a factor of 2–3 when moving from triple- to sextuple-ζ quality. The inclusion of diffuse (aug) functions has the most drastic effect, decreasing the error by a factor of roughly 6. As an example, comparing the cc-pVTZ and aug-cc-pVTZ basis sets, Δ̅_Basis_^MAE^ is reduced from ca. 1.2 to 0.2 eV upon inclusion of diffuse functions. Similarly for the NAOs, Δ̅_Basis_^MAE^ decreases by 0.62 eV comparing T2 to T2+aug2. While adding augmentation functions significantly impacts the satellite positions, the QP positions are barely affected (see SI Table S2). This suggests that augmentation functions are crucial for charge-neutral excitations but not for charged excitations. This observation aligns with previous TDDFT^110^ and Bethe–Salpeter equation studies,^105,106,110^ which found that adding augmentation functions to localized basis sets is essential for improving the description of the virtual space.

We note that the unaugmented ccX-*X*Z basis sets yield smaller Δ̅_Basis_^MAE^ values than the cc-pV*X*Z basis sets because they are larger by a factor of ca. 1.5–2. However, the performance of the ccX-*X*Z basis sets is significantly worse compared to any of the (smaller) augmented basis sets, underpinning the importance of very diffuse functions in the basis set.

The addition of core-optimized (C) functions in the aug-cc-pCV*X*Z basis sets does not improve Δ̅_Basis_^MAE^ compared to the aug-cc-pV*X*Z family. A similar trend is observed for the NAO basis sets T2+aug2+STO*X*: the inclusion of additional steep STO functions hardly affects Δ̅_Basis_^MAE^. This highlights that even for core-level satellites, satellite features depend only on the quality of the basis set in the valence space. However, we stress that core-optimized functions or additional STOs improve the accuracy of the 1s QP energies tremendously, decreasing the MAE with respect to the aug-cc-pCV6Z basis set from 1.1 to 0.1 eV going from T2+aug2 to T2+aug2+STO5 (see Table S2 in the SI).

For a balanced description of the full spectrum, including QPs and satellite features, we recommend using the aug-cc-pCV5Z basis set or T2+aug2+STO2 basis set, which both reliably reproduce satellite features within 0.1 eV of the aug-cc-pCV6Z reference. The NAO basis set is computationally significantly more efficient. As an example, for benzene, the T2+aug2+STO2 basis uses 468 basis functions, while the same calculation with an aug-cc-pCV5Z basis set has 1566 basis functions in total, increasing the computational cost by at least an order of magnitude. Therefore, will use the T2+aug2+STO2 basis set, unless stated otherwise.

### 6.4 Starting Point Dependence

In *GW* + *C*, the positions of the satellites relative to the QP peak are determined by the RPA excitation energies Ω^ν^, obtained by solving eq 6. The starting point enters the RPA equations through the orbital energy differences in the diagonal elements ϵ_*a*_ – ϵ_*i*_, while the off-diagonal (or coupling) elements in eq 7 are rather independent of the DFT functional as they only contain the direct Coulomb interactions (*ia*|*jb*). Here, we aim to assess what constitutes a reasonable starting points for RPA excitations.

To support the discussion, we list the lowest RPA excitation, Ω^1^, and the DFT gap between the HOMO and the lowest unoccupied molecular orbital (LUMO) for CH_4_ in Table 2, calculated using five different functionals. We include two DFT functionals without exact exchange, the local density approximation (LDA) and PBE, as well as two hybrid functionals, PBE0^111,112^ and PBEh (α = 0.45).^62,113^ The latter is identical to PBE0 but incorporates 45% exact exchange instead of 25%. Additionally, HF is included as the limiting case with full exact exchange. As reference, we report the lowest TDDFT excitation, Ω_TDDFT_^1^, which is known to provide excitation energies within 0.3 eV of experimental values for TDDFT@PBE0.^114,115^

**Table 2:** Fundamental DFT Gap, Δ_gap_^DFT^ = ϵ_LUMO_ – ϵ_HOMO_, Lowest RPA (Ω^1^) and TDDFT (Ω_TDDFT_^1^) Excitation Energies and Their Difference Δ*Ω*^1^ = Ω_TDDFT_^1^ – Ω^1^ for CH_4_ Using a cc-pVTZ Basis Set Computed with PySCF^86^ a

functional	Δ_gap_^DFT^	Ω^1^	Ω_TDDFT_^1^	Δ*Ω*^1^
LDA	9.70	10.12	9.91	–0.21
PBE	10.21	10.64	10.43	–0.21
PBE0	12.52	12.92	11.01	–1.91
PBEh (α = 0.45)	14.35	14.73	11.45	–3.28
HF	18.67	19.00	12.01	–6.99

a All values are in eV.

Across all functionals, the RPA excitation energies Ω^1^ closely match the HOMO–LUMO gap, indicating that the Coulomb coupling elements in eq 7 are small. Going from LDA to HF, Ω^1^ increases gradually with the amount of exact exchange by 9 eV. This enormous starting point dependence is mitigated in TDDFT, where exchange effects are added to eq 7 by including the exchange–correlation kernel *f*_*xc*_. Comparing LDA to HF, Ω_TDDFT_^1^ increases by only ≈2 eV. Looking at the difference between TDDFT and RPA results ΔΩ^1^ = Ω_TDDFT_^1^ – Ω^1^, LDA and PBE starting points produce RPA results close to TDDFT, with ΔΩ^1^ = −0.21. In contrast, for hybrid functionals and HF, the differences are substantial, with ΔΩ^1^ gradually changing from −2 to −7 eV. This shows that only DFT starting points without exact exchange, like PBE, yield RPA excitation energies of similar quality as TDDFT. This transfers also to the *GW* + *C* satellite spectra as shown in Figures S5 and S6 (SI). The spectral functions computed with hybrid starting points show nonphysical satellite spectra, lacking any resemblance with experiment.

These observations based on Table 2 can be generalized by looking at the physical meaning of orbital energy differences, like the HOMO–LUMO gap, in DFT based on nonhybrid functionals compared to HF. As discussed in detail in ref (116), the physical interpretation in both cases is different as consequence of different approximate mean-field potentials for virtual orbitals in HF and DFT theory. The HF mean-field potential of the virtual orbitals lacks an exchange hole, and thus the virtual orbitals are defined with respect to the field of *N* electrons, in difference to *N* – 1 electrons for the occupied orbitals.^116^ Consequently, HF virtual orbital energies approximate electron affinities in a reverted Koopmans’ theorem^87,116^ and orbital energy differences are no reasonable approximation to charge neutral excitations.^117^

In contrast, in DFT, virtual levels are computed for an (approximate) *N* – 1 electron potential which an excited electron would experience. Hence, DFT orbital energy differences resemble charge-neutral excitations, and RPA@PBE and RPA@LDA excitation energies are often a good approximation compared to TDDFT.^87,116^ Hybrid functionals interpolate between those limiting cases, and by increasing the amount of exact exchange the physical interpretation of the orbital energy differences changes toward the interpretation as fundamental gap. In TDDFT, the exchange contributions in *f*_*xc*_ counteract this effect, whereas RPA contains only Coulomb couplings which cannot adapt to this change.^118^

As the excitation energies of RPA@PBE resemble the TDDFT@PBE results, we can estimate the expected accuracy of *GW* + *C* satellites by comparison with previous TDDFT benchmarks: For TDDFT@PBE, the best results were obtained for π – π* excitations, with an MAE of 0.3 eV compared to experimental values,^114^ while other valence excitations (MAE = 0.6 eV) and Rydberg excitations (MAE = 0.8 eV) were predicted with much lower accuracy.^114,115^ Therefore, we expect *GW* + *C*@PBE to work well for organic molecules with conjugated π-systems where the dominant satellite features are due to π – π* transitions. Although hybrid functionals are known to provide improved TDDFT excitation energies with a broad range of applicability,^114^ their use in *GW* + *C* would demand to go beyond RPA by including additional terms in eq 6, which is effectively a vertex correction to the polarizability in *GW*.^119,120^

### 6.5 Shakeup Satellites in Molecules

**Figure 7:**
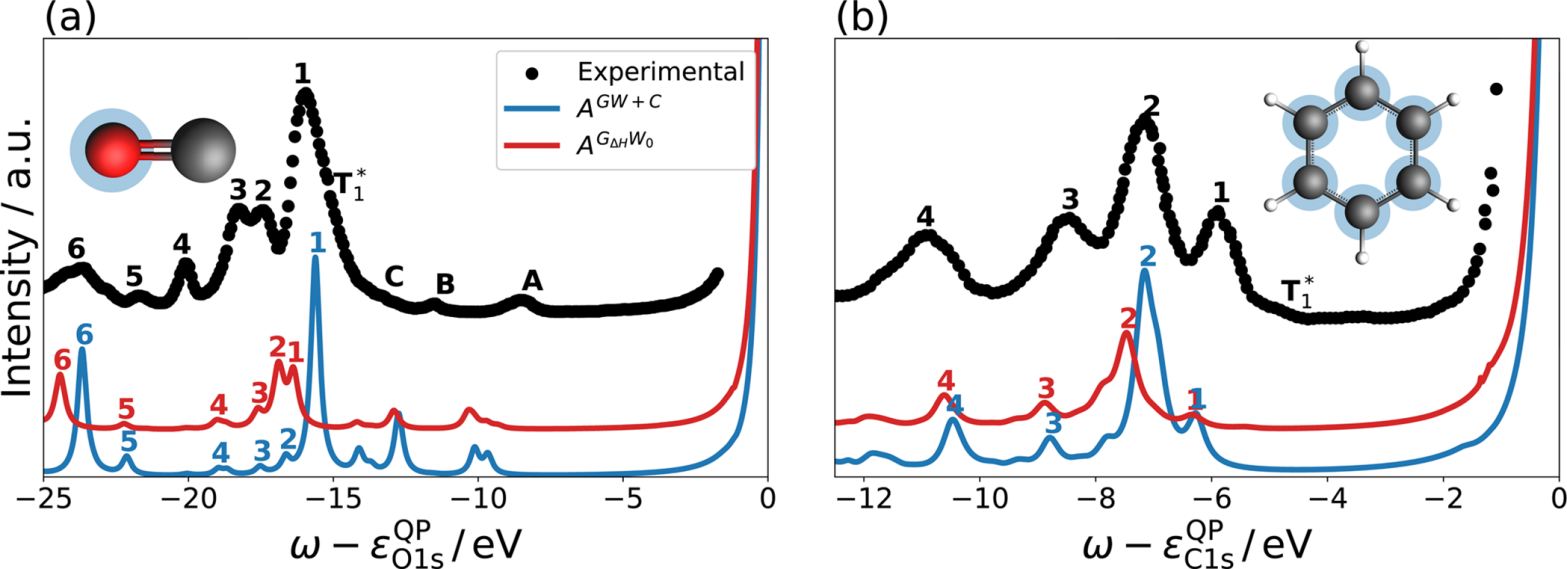
Comparison of the *G*_Δ*H*_*W*_0_ and *GW* + *C* spectral function (colored) to the gas-phase experiment (black) for carbon monoxide^30^ (a) and benzene (b).^121^ The peak assignment follows approximately the experiment, with numbers labeling singlet shakeup satellites with relevant intensity, while letters denote either inelastic losses (A, B, C) or triplet excitations (*T*_1_^*^).

In this work, we restrict the discussion to satellites associated with 1s excitations in molecules containing π-electrons. In Figure 7, we present our results for the O 1s level of carbon monoxide (CO) as a polarized and unconjugated system, and the C 1s level of benzene as an aromatic system, together with experimental gas-phase XPS data.^30,121^ Both CO^25,31,32^ and benzene^17,80,121,122^ have been the subject of several theoretical studies on core-level shakeup satellites, which we use here as reference. In addition, we provide spectral functions at the *G*_Δ*H*_*W*_0_ level to identify the effect of the vertex corrections in *GW* + *C*. Based on the findings in Sections 6.1 and 6.3, we use a PBE starting point with a T2+aug2+STO2 basis set and employ the decoupling approximation in combination with the CD-WAC algorithm. We point out that we compare the satellite region directly to the experiment by aligning the QP peaks and do not require a scissor shift for the satellite region.

#### 6.5.1 Comparison to Experiment

The experimental spectra are shown in black in Figure 7. The peaks are assigned as singlet (1–6) and triplet (*T*_1_^*^) shakeup satellites as well as inelastic losses (A–C), following the assignment in refs (30) and (121). We will focus on satellites due to singlet excitations, as *GW* + *C* excludes other types of excitations.

For CO, the O 1s shakeup spectrum has six singlet shakeup satellites in the low-energy region. The spectrum is dominated by the intense peak 1, which has been assigned to an admixture of π – π* and σ – σ* excitations by ADC,^31^ QDPTCI,^25^ and SAC–CI^32^ calculations. For the higher lying satellite peaks 2–5, the π – π* component decreases and is replaced by additional σ – σ* excitations, while peak 6 has been determined to be predominantly of Rydberg character.^25^ Besides the singlet excitations, several additional signals appear closer to the QP peak which are assigned to inelastic losses (A–C) and a triplet excitation *T*_1_^*^.^31,32^

In Figure 7a, *A*_O1s_^*GW*+*C*^ shows in general good agreement compared to the experimental spectrum. The integrated intensity of the satellite region is predicted as 16.9% of the QP peak, in excellent agreement with the experimental value of 18.9%. The position of the intense peak 1 is predicted within 0.25 eV of the experimental peak and correctly predicted to be the by far most intense shakeup state in the spectrum. For the peaks 2–5, the agreement of *A*_O1s_^*GW*+*C*^ is less satisfying, since the intensities are very low compared to the experiment, although the satellites appear within 0.5 eV of the reference. Peak 6 is again well predicted within 0.1 eV of the experimental counterpart, albeit the agreement might be somewhat fortunate as it previously has been assigned to a Rydberg excitation. Between −10 and −15 eV, several small peaks appear in *A*_O1s_^*GW*+*C*^, which approximately correspond to the signals (A–C) in the experimental spectrum. These peaks have previously been attributed to inelastic losses due to their pressure dependence,^30^ but they may also overlap with smaller singlet excitations.

For the benzene C 1s shakeup spectrum in Figure 7b, previous calculations assigned all peaks to singlet π – π* shakeup processes. The shakeup transitions 1 and 2 have been assigned to HOMO–LUMO transitions, which split into several levels due to symmetry reduction during the ionization process.^121^ Peak 3 and 4 are associated with a higher order π – π* transitions, although semiempirical configuration interaction calculations suggest substantial σ – σ* admixtures for peak 4.^17^ As for CO, a low-intensity triplet shakeup excitation is observed as shoulder of peak 1.

The *GW* + *C* spectral function agrees overall very well with the experimental spectrum for benzene, and the integrated relative intensity of the satellites matches the experimental value of 15% exactly. Peak 1 appears slightly too far from the QP peak in *GW* + *C*, but is still within 0.3 eV of the experimental reference. Peak 2 is calculated to be within 0.1 eV of the experimental signal and is correctly predicted as the most intense feature in the satellite spectrum. The *GW* + *C* spectral function exhibits several sharp features between −8 and −10 eV, therefore the assignment of the broad peak 3 is ambiguous. We assign peak 3 to the shakeup satellite with the highest intensity in this region, which is within 0.25 eV of the experimental signal. For peak 4, the assignment can again be done unambiguously. The calculated peak in the *GW* + *C* spectrum matches the experiment up to 0.5 eV, which is a slightly higher deviation compared to the satellites 1–3.

#### 6.5.2 Comparison with *G*_Δ*H*_**W**_0_

Comparing the *G*_Δ*H*_*W*_0_ and *GW* + *C* spectral functions, we observe strong differences in intensity and position of the satellite features. In general, in *G*_Δ*H*_*W*_0_, the spectral weight of the satellite region is only half of both experiment and *GW* + *C*, carrying only 9.2 and 8.8% of the QP peak intensity for CO and benzene, respectively. For CO, as an example, *G*_Δ*H*_*W*_0_ predicts peaks 1 and 2 to have similar magnitudes, which is in striking contrast to the experimental and *GW* + *C* results. However, the changes relative to *GW* + *C* are not systematic; some satellites, such as peak 2 in CO, are enhanced, while others, like peak 1 in CO, are suppressed.

Additionally, the satellites in *A*^*G*_Δ*H*_*W*_0_^ are shifted to higher binding energies compared to their *GW* + *C* counterparts. This effect is most pronounced for the intense peaks. For example, peaks 1 and 6 in CO shift by nearly 1 eV, while peak 2 in benzene shifts by approximately 0.4 eV. In contrast, less intense satellites show virtually no shift; for instance, peak 5 in CO appears almost at the same position in *G*_Δ*H*_*W*_0_ and *GW* + *C*. This is in line with the discussion in Sections 2.2 and 3.2, because satellites in all kinds of *GW* flavors appear at higher energies compared to the poles in the self-energy, while in *GW* + *C* the first-order satellites appear exactly at the poles of the *G*_Δ*H*_*W*_0_ self-energy, as evident by inspecting eqs 46 and 20. Therefore, we stress that the vertex corrections provided by *GW* + *C* approach are crucial for the interpretation of intense satellite features, within the boundaries derived in Section 3.2.

### 6.6 Acene Series

**Figure 8:**
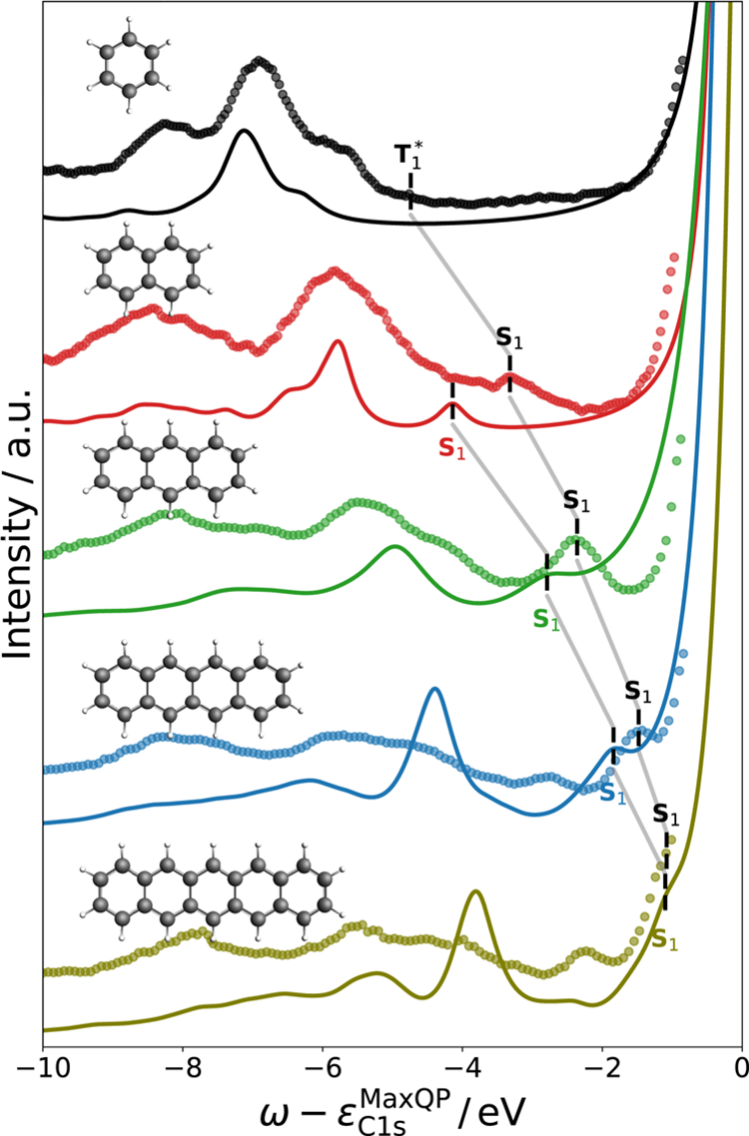
C 1s spectral functions *A*_C1s_^*GW*+*C*^ (solid line) of the acene series from benzene to pentacene compared to experimental XPS (dots) measured for multilayer films on Ag(111).^123^ The satellite spectrum is shown relative to the maximum of the main C 1s excitation. For each system, the position of the lowest singlet shakeup satellite (*S*_1_) is marked with a dashed line in the experiment (black) and the *GW* + *C* prediction (colored). For benzene, only the triplet component (*T*_1_) is observable.

We use the acene series with the general formula C_4*n*+2_ H_2*n*+4_ to demonstrate both the scalability the applicability of our *GW* + *C* implementation for the interpretation of shakeup features in molecules. In Figure 8, we compare *A*_C1s_^*GW*+*C*^(ω) with solid-state experimental XPS data from ref (123) for benzene (C_6_H_6_) to pentacene (C_22_H_14_). As before, the computed and measured spectra are aligned at the maximum of the QP peaks at ϵ_C1s_^MaxQP^. For systems with more than one inequivalent core-level, i.e., all acenes but benzene, ϵ_C1s_^MaxQP^ is a superposition of several C 1s peaks. Due to weak intermolecular interactions in molecular aggregates, the solid-state shakeup spectra are expected to be similar to the gas-phase spectra. However, in solid-state systems, additional effects such as extrinsic losses and vibrational broadening may modulate the intensity and peak shape. A comparison of the solid-state spectrum of benzene in Figure 8 with the gas-phase data in Figure 7 confirms that the primary difference is the increased peak broadening in the solid-state case.

The first satellite has been assigned to the lowest singlet (*S*_1_) excitation, which is dominated by a π – π* (HOMO–LUMO) transition. The *S*_1_ satellite appears in the spectra of all acenes besides benzene, where the respective peak is excluded for symmetry reasons and only the triplet component is visible.^123^ We define Δ^S_1_–MaxQP^ as separation between *S*_1_ and the maximum of the main line at ϵ_C1s_^MaxQP^ and list the experimental (Δ_Exp_^S_1_–MaxQP^) and theoretical (Δ_*GW*+*C*_^S_1_–MaxQP^) values in Table 3. With increasing chain length, we observe that Δ_Exp_^S_1_–MaxQP^ decreases by more than 2 eV going from naphthalene to pentacene, a trend which is is well reproduced by *GW* + *C*. The deviation of Δ_Exp_^S_1_–MaxQP^ and Δ_*GW*+*C*_^S_1_–MaxQP^ is in the range of 0.8–0.1 eV, gradually decreasing with the chain length.

**Table 3:** Experimental (Exp) and Theoretical (*GW* + *C*) QP-Satellite Splitting Δ^S_1_–MaxQP^ between the *S*_1_ Satellite and the Maximum of the C 1s Ionization Peak ϵ_C1s_^MaxQP^ for the Acene Series Compared to the Experimental Optical Gap and the Calculated Actual QP-Satellite Splitting Δ_*GW*+*C*_^S_1_–C_*x*_^ between the Satellite and the Generating Core-Level with the QP Energy ϵ_C_*x*__^QP^ a

	naphthalene	anthracene	tetracene	pentacene
optical gap	3.90^124^	3.12^125,126^	2.37^126,127^	1.85^126,128^
Δ_Exp_^S_1_–MaxQP^^123^	3.32	2.36	1.48	1.08
Δ_*GW*+*C*_^S_1_–MaxQP^	4.14	2.79	1.86	1.18
Δ_*GW*+*C*_^S_1_–C_*x*_^	4.15	2.97	2.16	1.60
ϵ_C_*x*__^QP^–ϵ_C1s_^MaxQP^	0.01	0.18	0.30	0.42

a All values in eV.

Beyond the *S*_1_ satellite, several broad, overlapping peaks appear between −4 and −8 eV. For benzene, naphthalene and anthracene, the most intense satellite is correctly predicted by *GW* + *C* and within 0.1–0.4 eV of the experiment. In the case of tetracene and pentacene, the position of the intense peak between −4 and −5 eV matches the reported experimental peaks equally well. However, the intensity of the satellite is overestimated in the *GW* + *C* spectral function, which might be explained by extrinsic effects like vibrations in the experiment.

In ref (123), it was expected that the lowest satellite excitation, i.e., Δ_Exp_^S_1_–MaxQP^, should coincide with the optical gap (see Table 3). However, experimentally it was found that Δ_Exp_^S_1_–MaxQP^ is substantially lower by 0.6–0.9 eV compared to the experimental optical gap. This puzzling effect was interpreted in terms of a reorganization of the valence shell upon ionization, which seems to become more important with increasing chain length. For pentacene, this effect was estimated to be as large as 0.8 eV, which accounts for roughly 50% of the optical gap. However, here we stress that for systems with more than one C 1s core-level, the interpretation of Δ_Exp_^S_1_–MaxQP^ as excitation energy is not accurate. Since both the satellite peak and the QP peak are aggregated from the sum of all nonequivalent C 1s levels to the spectral functions, the direct comparison of both makes the *intrinsic assumption that all core-levels contribute equally to the satellite spectrum*. In the following, we will demonstrate for pentacene that this is not the case.

**Figure 9:**
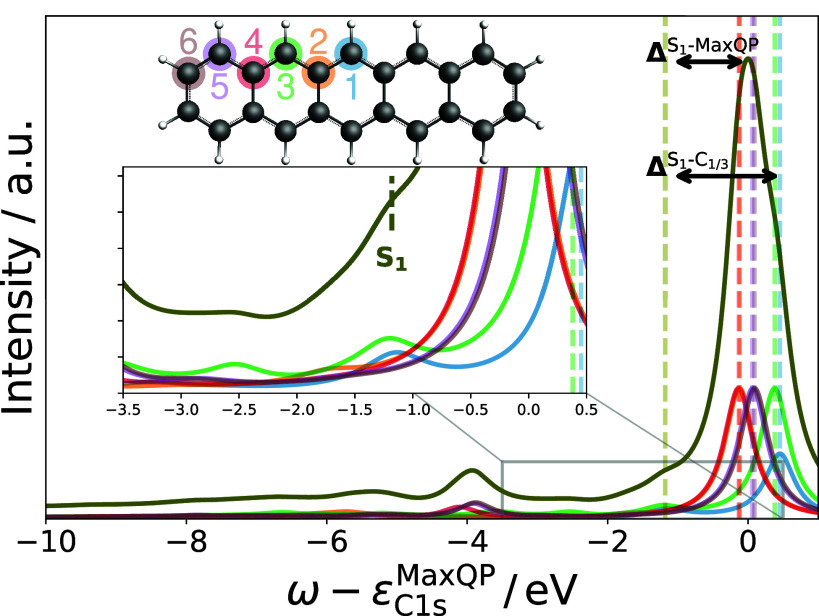
Contributions from all six inequivalent core-levels to the *GW* + *C* spectral function of pentacene. The inset highlights the satellite spectra of each individual core-level in the region of the *S*_1_ satellite.

In Figure 9, we resolve the six individual contributions C_1–6_ to the C 1s spectral function of pentacene. For each individual core-level C_*x*_, the distance Δ^S_1_–C_*x*_^ equals Ω^1^, which is the optical gap in the RPA approximation. By looking at the contributions from the individual core-levels C_1_–C_6_, we observe that most of the intensity of the *S*_1_ satellite is contributed by the C 1s levels C_1_ and C_3_. The other core-levels (C_2_, C_4_–C_6_) couple only weakly to the *S*_1_ excitation, and therefore the position of the *S*_1_ satellite in the aggregated spectral function has to be interpreted relative to the C_1_ and C_3_ QP peaks at ϵ_C_1/3__^QP^ and not to the maximum at ϵ_C1s_^MaxQP^. Since the C_1_ and C_3_ peaks at ϵ_C_1/3__^QP^ appear at lower binding energies than the maximum of the QP, the *S*_1_ satellite appears shifted by ϵ_C_1/3__^QP^–ϵ_C1s_^MaxQP^ ≈ 0.42 eV. By adding this shift to Δ_Exp_^S_1_–MaxQP^, the difference between the optical gap and the satellite excitation energy is decreased by more than half to only 0.35 eV.

This example highlights that in systems with several inequivalent core-level the exact knowledge of the generating level(s) is mandatory for the interpretation of shakeup satellites. This kind of knowledge is provided by *GW* + *C*, and can be directly applied to interpret both gas-phase and solid-state XPS spectra of molecules.

We point out that the system size presented here is still far from the actual computational limit of our method. For pentacene, the largest system in this study with 22 core states and 1600 basis functions in total, the computation took ca. 5600 CPU hours. Based on the results presented in ref (76), we expect our *GW* + *C* implementation to be applicable to systems with up to 100 atoms and even beyond, depending on the number of core-levels and the basis set.

## 7 Conclusions

In this work, we presented a scalable *GW* + *C* implementation for the prediction of molecular core-level satellites. Building on ref (76), we combined *GW* + *C* with the CD-WAC approach in an efficient all-electron NAO framework, enabling the calculation of core-level spectral functions with *N*^4^ scaling for systems with more than 100 atoms. We derived several key recommendations for calculating core-level spectral functions with *GW* + *C*: (i) The decoupling approximation is essential for localized basis sets; (ii) The T2+aug2 basis set is suitable for accurate satellite properties, with additional core-level STOs improving QP energies; (iii) A PBE (GGA) starting point ensures reliable satellite properties. We tested our computational framework for CO and benzene and the acene series up to pentacene, yielding an agreement of roughly 0.5 eV for the dominant satellite features and correctly predicting qualitative trends. For pentacene, we demonstrated how *GW* + *C* spectral functions can be a valuable tool for the interpretation of experimental shakeup spectra, paving the way for future applications.

Our ongoing and future work includes incorporating additional vertex corrections, e.g. by making use of recent developments connecting *GW* and coupled cluster theory.^27,129−131^ We also aim to develop a nonlinear extension of the *GW* + *C* method to avoid nonphysical contributions in the spectral function in all cases. Furthermore, we plan to extend our approach to *p*- and *d*-levels of heavy elements by adapting a fully relativistic two-component *G*_0_*W*_0_ scheme^132−134^ as starting point, which is currently under development.

## Supporting Information Available

*G*_Δ*H*_*W*_0_ core-level binding energies are provided for the CORE65 benchmark set for all 22 basis sets (TXT)
Discussion of the *G*_Δ*H*_*W*_0_ QP peak and a detailed derivation of eq 64; comparison of the CD-WAC fit for diagonal and off-diagonal matrix elements; technical description of our satellite selection criterion used for the calculation of eq 73; Tables S1 and S2 include statistics for the satellite- and quasiparticle energies for the CORE65 benchmark set*; GW* + *C* spectral functions based on starting points with various amount of exact exchange for CH_4_ and CO (PDF)
