# Supplementary material for: GW Plus Cumulant Approach for Predicting Core-Level Shakeup Satellites in Large Molecules
Source: J Chem Theory Comput. 2025 Mar 3;21(6):3101–19. doi: 10.1021/acs.jctc.4c01754 (PMC11948339; doi:10.1021/acs.jctc.4c01754)
Supplement: Supplementary file 2 — ct4c01754_si_002.pdf [file ct4c01754_si_002.pdf]

Supporting Information: *GW* plus cumulant approach for predicting core-level shake-up satellites in large molecules.

Jannis Kockläuner* and Dorothea Golze*

Faculty of Chemistry and Food Chemistry, Technische Universität Dresden, 01062 Dresden, Germany

E-mail: jannis.kocklaeuner@tu-dresden.de; dorothea.golze@tu-dresden.de

S1 Equivalence of $GW + C$ and $G_{\Delta H}W_0$ quasiparticle energies

In $GW + C$, the quasiparticle energy is defined as

$$\epsilon_n^{\mathrm{QP}} = \epsilon_n + \Delta H_n \tag{S1}$$

with ΔH_n being the level specific Hedin shift defined in Eq. (19) in the main text. In $G_{\Delta H}W_0$, the quasiparticle energy is obtained as solution of the non-linear quasiparticle equation

$$\epsilon_n^{\mathrm{QP}} = \epsilon_n + \Sigma_n^{x,G_0W_0} - v_n^{xc} + \Sigma_n^{c,G_0W_0}(\epsilon_n^{\mathrm{QP}} - \Delta H_n) \tag{S2}$$

By plugging Eq. (S1) as ϵ_n^{QP} into Eq. (S2), we obtain

$$\begin{aligned}\epsilon_n + \Delta H_n &= \epsilon_n + \Sigma_n^{x,G_0W_0} - v_n^{xc} + \Sigma_n^{c,G_0W_0}(\epsilon_n + \Delta H_n - \Delta H_n) \\ &= \epsilon_n + \Sigma_n^{x,G_0W_0} - v_n^{xc} + \Sigma_n^{c,G_0W_0}(\epsilon_n) \\ &= \epsilon_n + \Delta H_n\end{aligned} \tag{S3}$$

Since the left- and right-hand side of Eq. (S3) are equal, the $GW + C$ solution in Eq. (S1) solves the quasiparticle equation in Eq. (S2).

S2 Taylor expansion of the $GW + C$ satellite spectrum

We start by defining

$$e^{\tilde{C}_n^c(t)} = Z_n e^{C_n^{\mathrm{S}}(t)} \tag{S4}$$

where

$$Z_n = \exp\left(-\frac{1}{\pi}\int \mathrm{d}\omega \frac{\left|\mathrm{Im}\,\Sigma_n^{c,G_0W_0}(\omega + \epsilon_n)\right|}{\omega^2}\right) \tag{S5}$$

and

$$C_n^{\mathrm{S}}(t) = \int \mathrm{d}\omega \frac{1}{\pi}\frac{\left|\mathrm{Im}\,\Sigma_n^{c,G_0W_0}(\omega + \epsilon_n)\right|}{\omega^2} e^{-\mathrm{i}\omega t} \tag{S6}$$

we use a Taylor expansion of $e^{C_n^{\mathrm{S}}(t)}$ as

$$e^{C_n^{\mathrm{S}}(t)} \approx 1 + \int \mathrm{d}\omega \frac{1}{\pi}\frac{\left|\mathrm{Im}\,\Sigma_n^{c,G_0W_0}(\omega + \epsilon_n)\right|}{\omega^2} e^{-\mathrm{i}\omega t} + O(n^2) \tag{S7}$$

The second term in Eq. (S7) is a Fourier transform from frequency- to time space, the frequency-dependent term is obtained as

$$e^{C_n^{\mathrm{S}}(\omega)} \approx 1 + \frac{1}{\pi}\frac{\left|\mathrm{Im}\,\Sigma_n^{c,G_0W_0}(\omega + \epsilon_n)\right|}{\omega^2} + O(n^2) \tag{S8}$$

Plugging Eq. (S8) in Eq. 62 (main text) yields

$$G_n^C(\omega) \approx \frac{Z_n}{\pi}\left(G_{0,n}^{\mathrm{QP}}(\omega) + \frac{1}{\pi}\int \mathrm{d}\omega' G_{0,n}^{\mathrm{QP}}(\omega+\omega')\frac{\left|\mathrm{Im}\,\Sigma_n^{c,G_0W_0}(\omega'+\epsilon_n)\right|}{\omega'^2} + O(n^2)\right) \tag{S9}$$

Approximating $-\mathrm{Im}\,G_{0,n}^{\mathrm{QP}}$ as $\delta(\omega-\epsilon_n^{\mathrm{QP}})$ and carrying out the convolution, we obtain an approximate expression for the spectral function.

$$A_n^{GW+C}(\omega) \approx \frac{Z_n}{\pi}\left(\delta\left(\omega-\epsilon_n^{\mathrm{QP}}\right) + \frac{1}{\pi}\frac{\left|\mathrm{Im}\,\Sigma_n^{c,G_0W_0}(\omega-\Delta H_n)\right|}{\left(\omega-\epsilon_n^{\mathrm{QP}}\right)^2} + O(n^2)\right) \tag{S10}$$

The Delta function in Eq. (S10) creates the quasiparticle peak, while $\mathrm{Im}\,\Sigma_{\mathrm{n}}^{c,G_0W_0}(\omega-\Delta H_n)$ contains the single excitations and creates the satellites in the spectral function. The shift ΔH_n is responsible for placing the satellites in the spectral function relative to the quasiparticle peak, as the peaks in $\mathrm{Im}\,\Sigma_{\mathrm{n}}^{c,G_0W_0}(\omega)$ appear relative to DFT eigenvalues.[S1,S2] To first order, Eq. (S10) can be rewritten in terms of $\mathrm{Im}\,\Sigma_{\mathrm{n}}^{c,G_{\Delta H}W_0}(\omega)$ as

$$A_n^{GW+C}(\omega) \approx \frac{Z_n}{\pi}\left(\delta\left(\omega-\epsilon_n^{\mathrm{QP}}\right) + \frac{\left|\mathrm{Im}\,\Sigma_n^{c,G_{\Delta H}W_0}(\omega)\right|}{\pi\left(\omega-\epsilon_n^{\mathrm{QP}}\right)^2}\right) \tag{S11}$$

S3 Satellite selection

In order to determine the basis set convergence of the relative satellite positions $\Delta^{\mathrm{Sat1-QP}}$ in Section 6.3, we require an automated workflow to determine $\Delta^{\mathrm{Sat1-QP}}$ for in total 1430 spectra. As we work with a numerical approach, the RPA energies Ω^ν are not directly accessible, only the spectral function $A_n^{GW+C}(\omega)$. To determine $\Delta^{\mathrm{Sat1-QP}}$, we cannot make use of the analytical expressions in Eq. 46 (main text), but require a predefined criterion to assign the satellite in question unambiguously across all systems and satellites.

We are only interested in satellites that carry spectral weight, therefore we use the integrated intensity of the spectral function as criterion: The position ϵ^{Sat1} used for determining $\Delta^{\mathrm{Sat1-QP}}$ is

defined by the position were the integrated intensity outside of the quasiparticle peak reaches 0.1 % of the main peak intensity. The resulting peak positions are shown in Figure S1, where in every case the onset of the first satellite with spectral weight is reliably found. We note that the obtained positions might not directly coincide with $\epsilon_n^{\mathrm{QP}} - \Omega^{\nu}$, because the criterion is not necessarily fulfilled at the peak maxima. This underlines that the chosen criterion includes both position and intensity of the satellite. Such an approach is required here, as we are not interested in states that do not carry any spectral weight, but only in satellites that actually couple to the core-level.

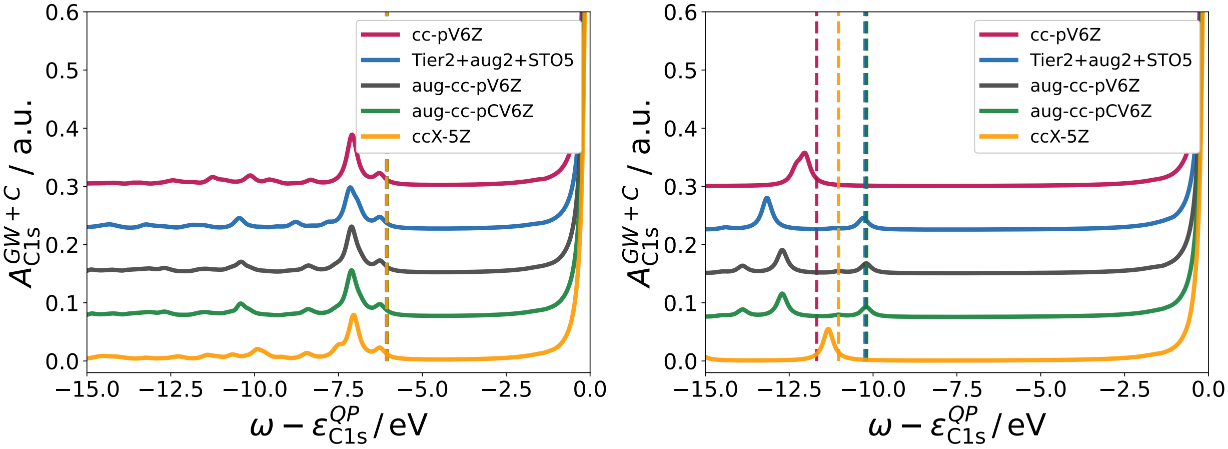

Figure S1: Automated satellite selection based on the intensity criterion described in the text using the largest basis set of each family for CH_4 (left) and C_6H_6 (right). For each basis set, the chosen ϵ^{Sat1} for the calculation of $\Delta^{\mathrm{Sat1-QP}}$ is indicated by a dashed line.

For the CORE65 benchmark set, we report the resulting mean average errors ($\bar{\Delta}_{\mathrm{Basis}}^{\mathrm{MAE}}$), mean errors (ME) and the standard deviation (Dev.) with respect to the aug-cc-pCV6Z basis set in Table S1 for all 22 basis sets.

S4 CD-WAC fit for (off-)diagonal elements of the screened interaction

Here, we demonstrate the accuracy of the CD-WAC fit for both diagonal- and off-diagonal elements of $W_{mn}^{c}(\omega)$ by reproducing Figure 4b) in the main text using the CD-WAC approximation. In Figure S2, we show $\mathrm{Im}\, W_{\mathrm{C1s},1-5}^{c}(\omega)$ for CH_4 in a cc-pV6Z basis, reproducing both the shake-up features in $\mathrm{Im}\, W_{\mathrm{C1s,C1s}}^{c}(\omega)$ and the correlation satellites in $\mathrm{Im}\, W_{\mathrm{C1s},2-5}^{c}(\omega)$ accurately. Therefore, we

Table S1: Basis set convergence with respect to the aug-cc-pCV6Z basis set as measured by the onset of the satellite region $\Delta_{\mathrm{Sat1-QP}}$, averaged using all 65 core-levels of the CORE65 benchmark set.

Basis Set	$\bar{\Delta}^{\mathrm{MAE}}_{\mathrm{Basis}}$ / eV	ME / eV	Dev. / eV
cc-pVTZ	1.25	-1.25	1.14
cc-pVQZ	1.04	-1.04	1.07
cc-pV5Z	0.72	-0.71	0.81
cc-pV6Z	0.52	-0.51	0.62
T2	0.73	-0.72	0.97
T2+aug2	0.11	-0.06	0.16
T2+aug2+STO1	0.10	-0.07	0.15
T2+aug2+STO2	0.10	-0.06	0.15
T2+aug2+STO3	0.09	-0.06	0.14
T2+aug2+STO4	0.10	-0.06	0.14
T2+aug2+STO5	0.09	-0.06	0.13
aug-cc-pVTZ	0.23	-0.17	0.32
aug-cc-pVQZ	0.16	-0.11	0.22
aug-cc-pV5Z	0.09	-0.06	0.15
aug-cc-pV6Z	0.02	0.00	0.04
aug-cc-pCVTZ	0.23	-0.16	0.33
aug-cc-pCVQZ	0.16	-0.11	0.22
aug-cc-pCV5Z	0.10	-0.06	0.14
aug-cc-pCV6Z	0.00	0.00	0.00
ccX-TZ	0.69	-0.69	0.68
ccX-QZ	0.42	-0.39	0.54
ccX-5Z	0.31	-0.24	0.40

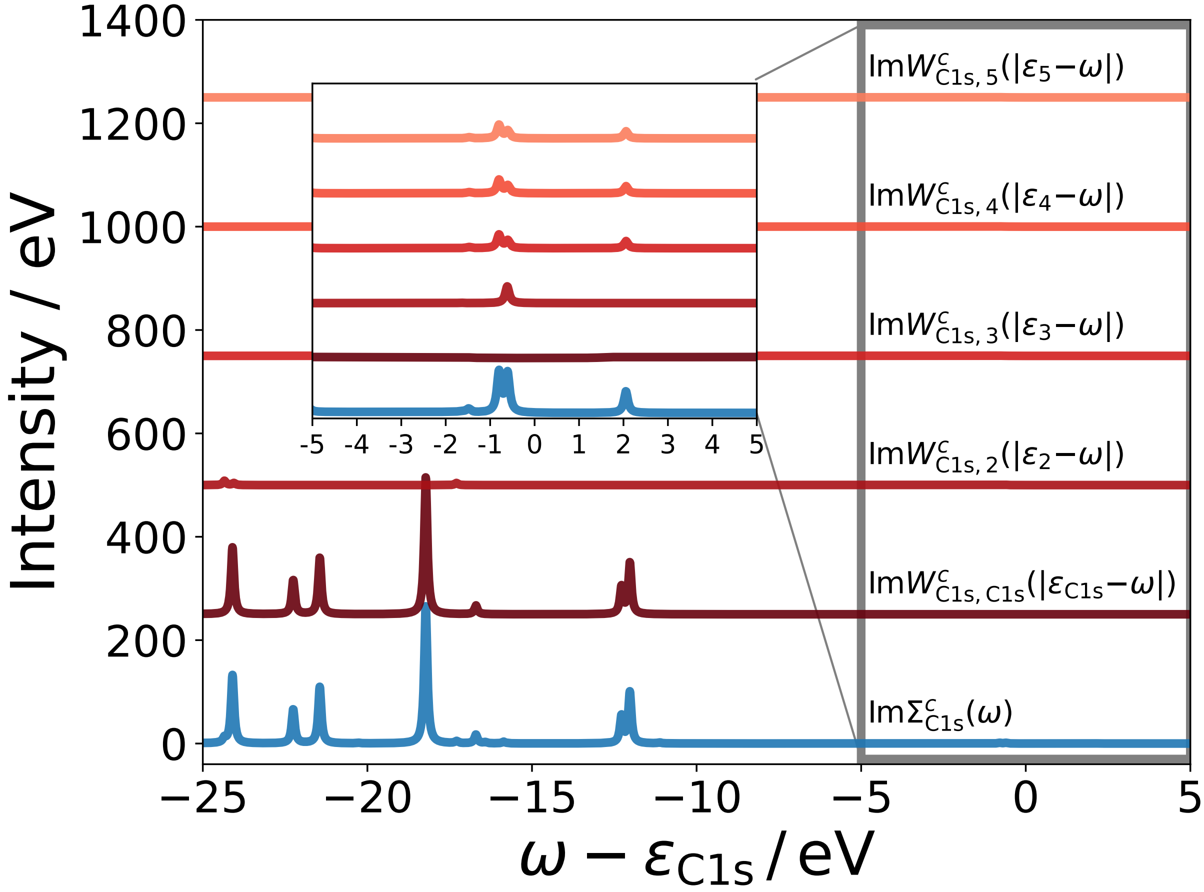

Figure S2: Imaginary part of the CD-WAC fit of the diagonal- and off-diagonal elements of the screened interaction $\mathrm{Im}\, W^c_{mn}(\omega)$ and the resulting self-energy $\mathrm{Im}\, \Sigma^{c,G_0W_0}_{\mathrm{C1s}}$ for CH_4 in a cc-pV6Z basis.

conclude that the CD-WAC approximation cannot be used to avoid spurious correlation satellites, as such satellites are equally well approximated as the physical shake-up states.

S5 Basis set convergence of $G_{\Delta H}W_0$ quasiparticle energies

Here, we compare core level binding energies (CLBE's), defined as $-\epsilon_n^{\mathrm{QP}}$, for the CORE65 benchmark with respect to experimental and to the aug-cc-pCV6Z references. All values were calculated using a CD-WAC self-energy with the settings defined in the main text. The mean absolute errors (MAE) and mean errors (ME) of the CLBE's on the $G_{\Delta H}W_0$@PBE are given in Table S1. Following Ref. S3, we add the following relativistic corrections to the CLBE to improve comparison with experimental data: 0.1176 eV for C1s, 0.2355 eV for N1s, 0.4244 eV for O1s and 0.7080 eV for F1s. For all basis set, we provide the statistical distribution of the errors with respect to the experiment in Figure S3 and with respect to the aug-cc-pCV6Z basis set in Figure S4.

S6 Starting point dependence of the $GW + C$ spectral function

Here, we demonstrate that the use of starting points with a certain amount of non-local exchange effects results in a static shift of the satellite spectrum, as discussed in section 6.4. For this sake, we computed $A_{\mathrm{C1s}}^{GW+C}(\omega)$ for CH_4 and $A_{\mathrm{O1s}}^{GW+C}(\omega)$ for CO using four different starting points: i) The PBE[S5] GGA functional ii) The PBE0[S6,S7] hybrid functional with 25 % HF-exchange iii) The PBEh(α=45)[S3] hybrid functional with 45 % HF-exchange and iv) The HF approximation. The resulting spectral functions are displayed in Figures S5 and S6.

For both CH_4 and CO, the satellite region is shifted by a static shift of roughly -10 eV going from PBE to HF. Despite the strong shift, the number and intensities of the satellites remain similar, although the intensity decreases slightly with increasing amount of exact exchange. We note that for CH_4 the shift is not exactly equal to the 9 eV difference reported in Table 2 in section 6.4 of the main text. This is because the first satellite in Figure S5 does not correspond to the excitation Ω^1, but a higher excitation Ω^{Sat1}, as Ω^1 carries no intensity in the spectral function. Comparing

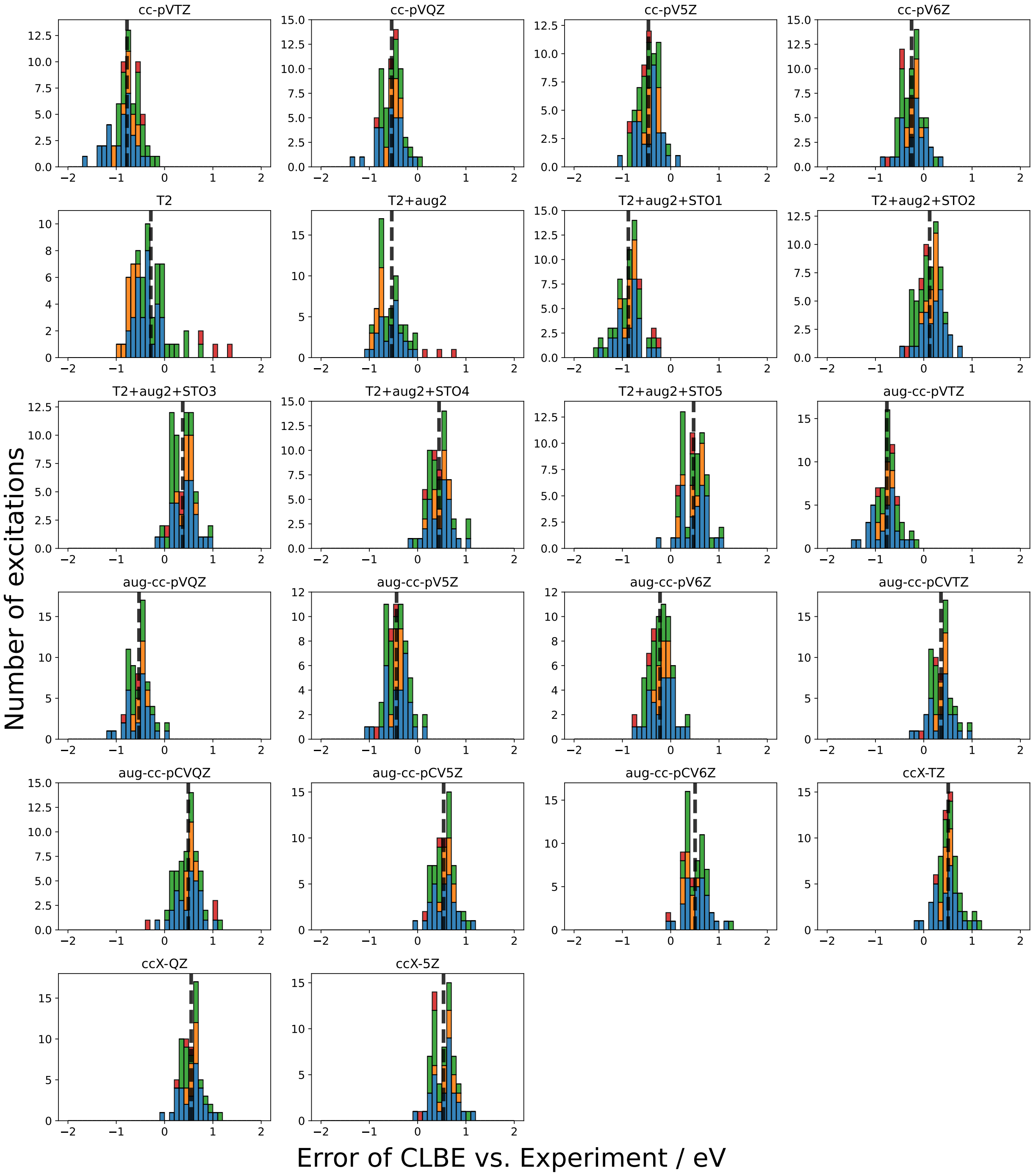

Figure S3: Distribution of errors with respect to the experiment for absolute 1s CLBEs of the CORE65 benchmark set for 22 different basis sets. The error is defined as $\text{error}_i = \text{CLBE}_{i,G_{\Delta H}W_0} - \text{CLBE}_{i,\text{Ref}}$. Relativistic effects are included following Ref S3. Colors indicate different core-levels, i.e. C1s (blue), N1s (orange), O1s (green) and F1s (red).

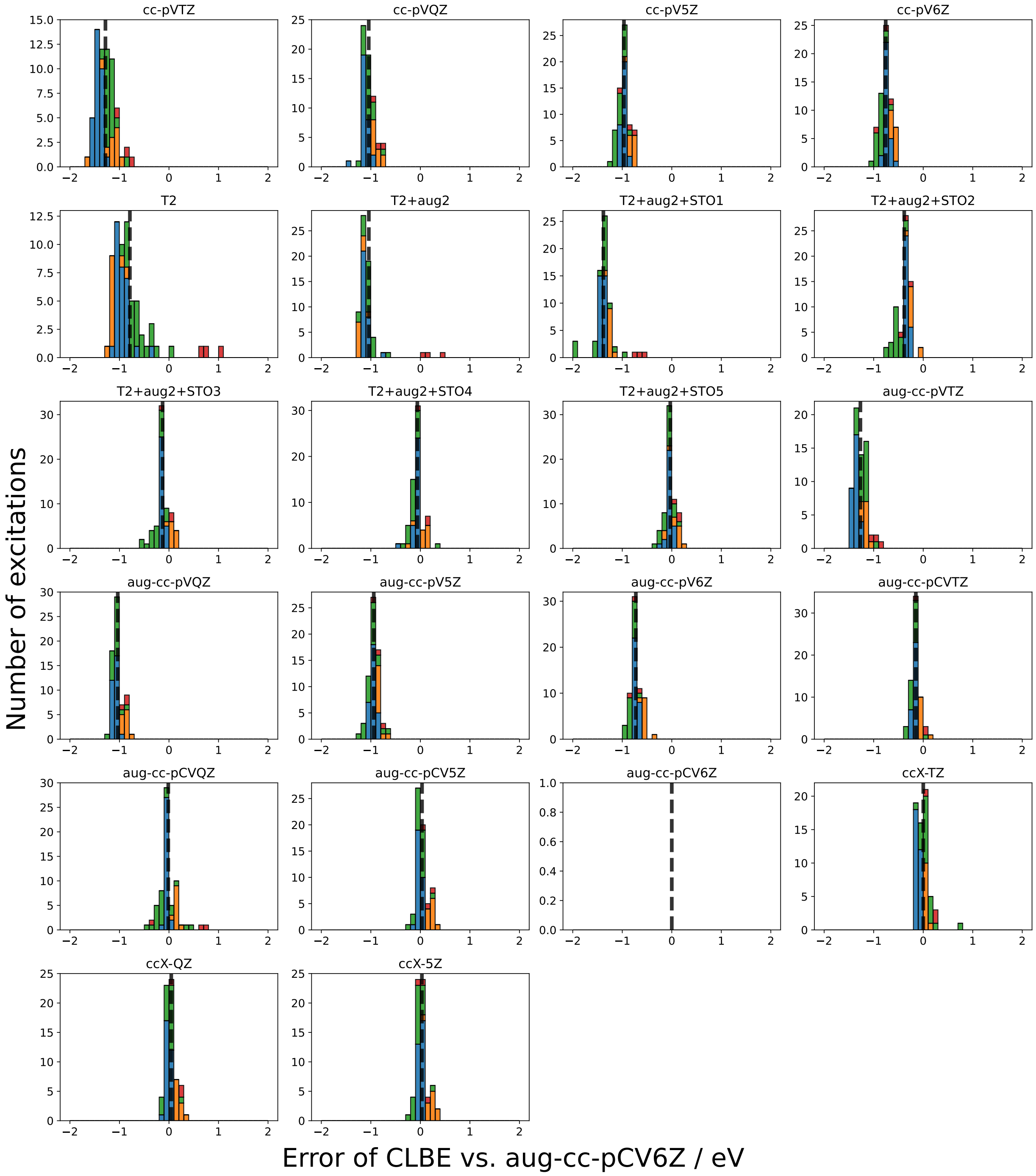

Figure S4: Distribution of errors with respect to the experiment for absolute 1s CLBEs of the CORE65 benchmark set for 22 different basis sets. The error is defined as $\text{error}_i = \text{CLBE}_{i,G_{\Delta H}W_0} - \text{CLBE}_{i,\text{Ref}}$. Colors indicate different core-levels, i.e. C1s (blue), N1s (orange), O1s (green) and F1s (red).

Table S2: Statistics of $G_{\Delta H}W_0$ core level binding energies for the CORE65 benchmark set relative to experimental and aug-cc-pCV6Z results.

Basis Set	vs. Experiment		vs. aug-cc-pCV6Z	
	MAE / eV	ME / eV	MAE / eV	ME / eV
cc-pVTZ	0.78	-0.78	1.28	-1.28
cc-pVQZ	0.54	-0.54	1.04	-1.04
cc-pV5Z	0.46	-0.46	0.97	-0.97
cc-pV6Z	0.29	-0.25	0.76	-0.76
Tier2	0.44	-0.28	0.87	-0.79
sto0	0.58	-0.54	1.06	-1.04
sto1	0.88	-0.88	1.38	-1.38
sto2	0.22	0.12	0.38	-0.38
sto3	0.38	0.38	0.16	-0.13
sto4	0.45	0.44	0.11	-0.06
sto5	0.48	0.47	0.09	-0.03
aug-cc-pVTZ	0.76	-0.76	1.27	-1.27
aug-cc-pVQZ	0.53	-0.53	1.03	-1.03
aug-cc-pV5Z	0.44	-0.44	0.94	-0.94
aug-cc-pV6Z	0.26	-0.22	0.73	-0.73
aug-cc-pCVTZ	0.37	0.36	0.16	-0.15
aug-cc-pCVQZ	0.50	0.49	0.14	-0.01
aug-cc-pCV5Z	0.54	0.54	0.08	0.03
aug-cc-pCV6Z	0.51	0.50	0.00	0.00
ccX-3Z	0.51	0.51	0.10	0.00
ccX-4Z	0.55	0.55	0.07	0.05
ccX-5Z	0.54	0.54	0.08	0.03

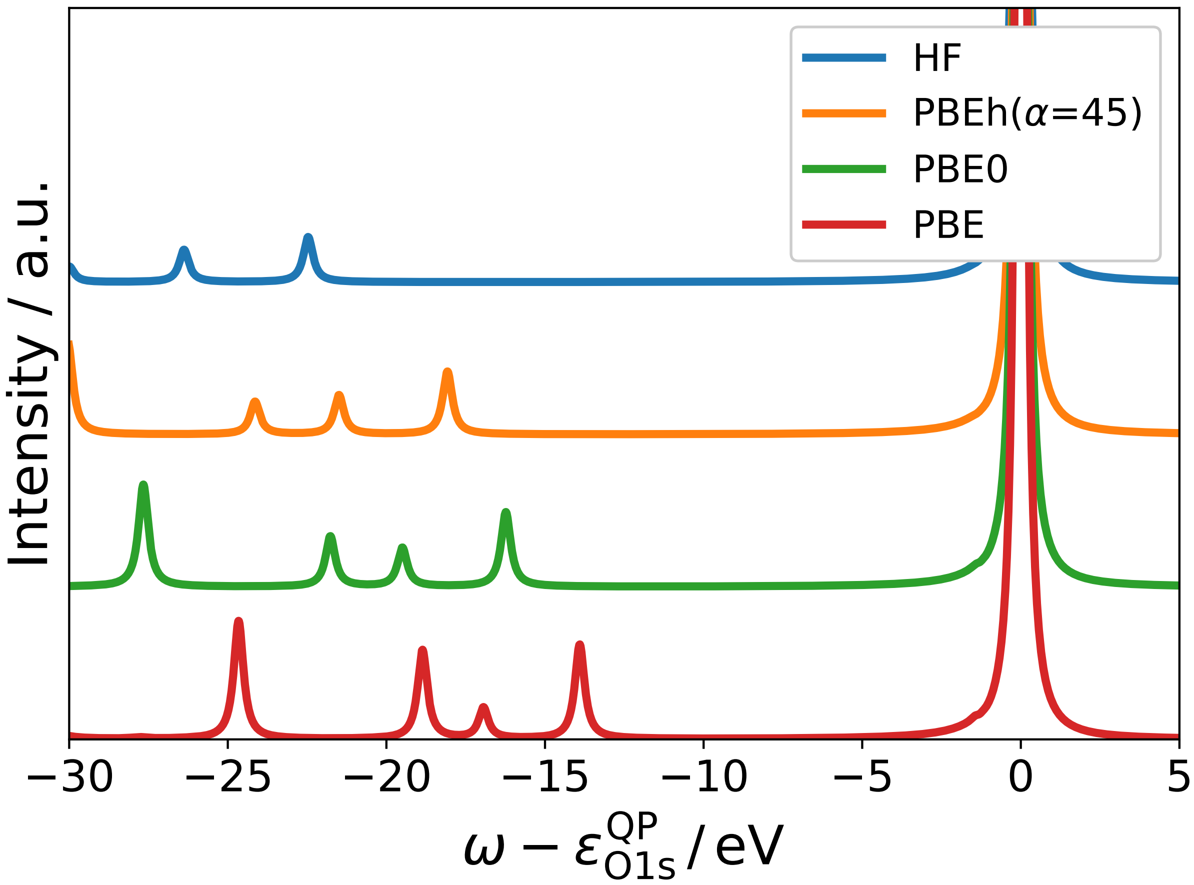

Figure S5: $A_{\mathrm{C1s}}^{GW+C}(\omega)$ of CH_4 (cc-pVTZ basis) computed with different amounts of HF-exchange.

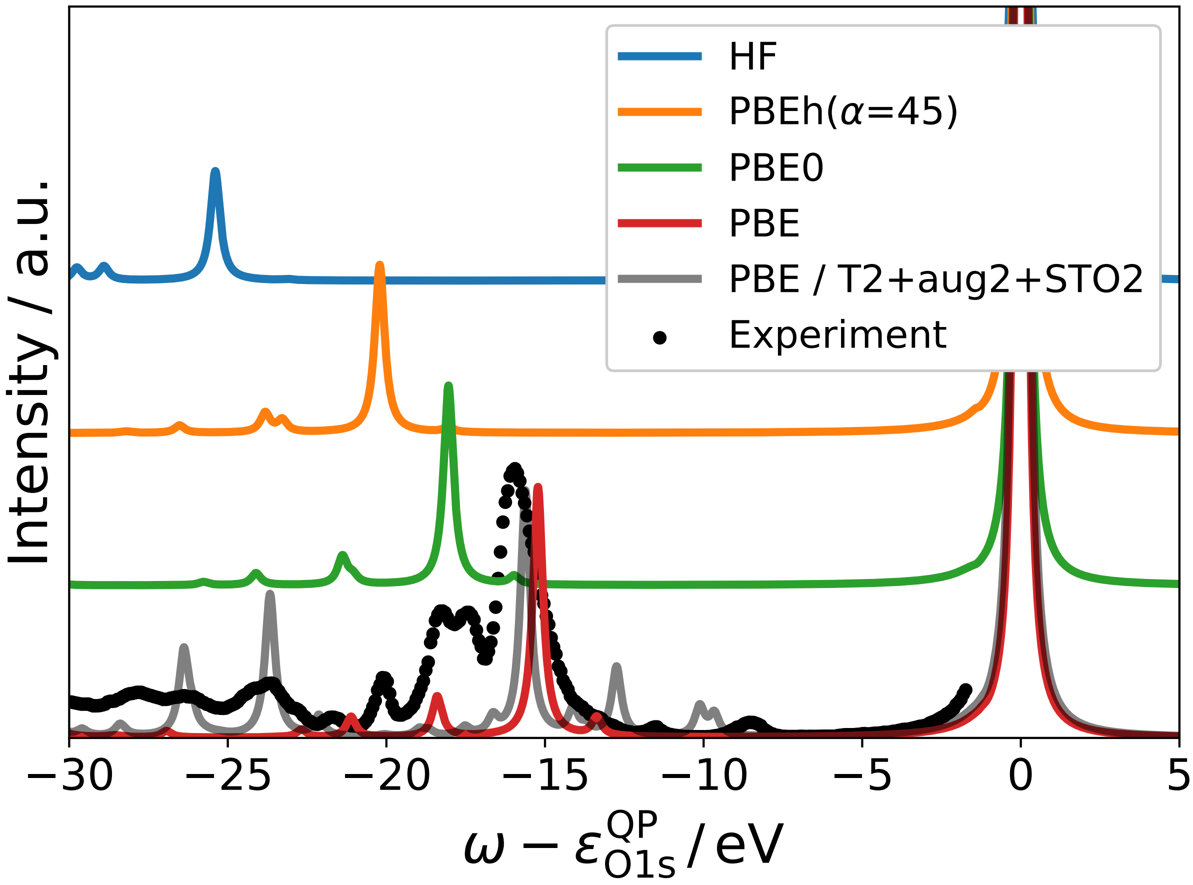

Figure S6: $A^{GW+C}_{\mathrm{O1s}}(\omega)$ of CO (cc-pVTZ basis) computed with different amounts of HF-exchange compared to the experiment.[S4] The T2+aug2+STO2 spectral function is shown as well to facilitate comparison with the main text.

$A_{\mathrm{O1s}}^{GW+C}(\omega)$ to the experiment in Figure S6, we note that the agreement worsens with increasing amount of exact exchange.

References

[S1] Aryasetiawan, F.; Hedin, L.; Karlsson, K. Multiple plasmon satellites in Na and Al spectral functions from ab initio cumulant expansion. *Phys. Rev. Lett.* **1996**, *77*, 2268.

[S2] Vila, F. D.; Kas, J. J.; Rehr, J. J.; Kowalski, K.; Peng, B. Equation-of-Motion Coupled-Cluster Cumulant Green's Function for Excited States and X-Ray Spectra. *Front. Chem.* **2021**, *9*, 734945.

[S3] Golze, D.; Keller, L.; Rinke, P. Accurate absolute and relative core-level binding energies from GW. *J. Phys. Chem. Lett.* **2020**, *11*, 1840–1847.

[S4] Schirmer, J.; Angonoa, G.; Svensson, S.; Nordfors, D.; Gelius, U. High-energy photoelectron C 1s and O 1s shake-up spectra of CO. *J. Phys. B* **1987**, *20*, 6031.

[S5] Perdew, J. P.; Burke, K.; Ernzerhof, M. Generalized gradient approximation made simple. *Phys. Rev. Lett.* **1996**, *77*, 3865.

[S6] Adamo, C.; Barone, V. Toward reliable density functional methods without adjustable parameters: The PBE0 model. *J. Chem. Phys.* **1999**, *110*, 6158–6170.

[S7] Ernzerhof, M.; Scuseria, G. E. Assessment of the Perdew–Burke–Ernzerhof exchange-correlation functional. *J. Chem. Phys.* **1999**, *110*, 5029–5036.
